# Organic material mulching regulated core microbial groups to promote soil carbon and nitrogen cycling and improve faba bean productivity under a triple-cropping system in purple soil hilly region of southwest China

**DOI:** 10.3389/fmicb.2025.1602633

**Published:** 2025-07-16

**Authors:** Ke Ren, Wenfeng Song, Zehui Wei, Lixia Song, Ming Liu, Yuling Zhou, Yuzhuo Zhen, Xinyao Wu, Kaiyuan Gu, Khanom Simarani, Longchang Wang

**Affiliations:** ^1^College of Agronomy and Biotechnology Southwest University Engineering Research Center of South Upland Agriculture, Ministry of Education, Chongqing, China; ^2^Division of Microbiology, Institute of Biological Sciences, Faculty of Science, Universiti Malaya, Kuala Lumpur, Malaysia

**Keywords:** straw mulch, milk vetch, soil carbon cycle, molecular ecological networks, faba bean productivity

## Abstract

**Background:**

Intensive agricultural production leads to the reduction of soil carbon (C) and nitrogen (N) reserves, and organic material mulching (OMM) can improve microbial community structure and promote C and N accumulation. The multi-cropping system based on legumes can provide abundant organic mulching material and improve soil quality, but the mechanism by which OMM provides ecological benefits via C and N cycling in this system is still unclear.

**Methods:**

In this study, a field experiment of organic mulch under the triple-cropping system of faba bean (*Vicia faba* L.)-corn (*Zea mays* L.)-sweet potato (*Ipomoea batatas* Lam.) was performed. Four treatments were set up: without organic material mulching (CK), straw mulching (S), milk vetch (*Astragalus sinicus* L.) mulching (M), and straw and milk vetch mulching (SM). The dynamic changes in soil aggregates, C and N contents, enzyme activities, microbial communities and faba bean productivity under organic mulching were studied for 2 years (from October 2020 to May 2022).

**Results:**

The results demonstrated that supplementation of OMM (S, M, and SM) significantly improved the stability of soil aggregates, the nutrient (C and N) contents, and the activities of acquiring enzymes compared to CK. OMM promoted the establishment of key microbial communities dominated by Actinobacteria, Bacteroidetes, Ascomycota, and Basidiomycota. Predicted functional profiles based on PICRUSt and FUNGuild analyses suggest possible upregulation of up-regulated genetic information processing, metabolism, and organismal systems functional pathways. Additionally, the enhancement of soil C and N cycling efficiency may be associated with an increase in the proportion of saprotrophs and symbiotrophs. Both the random forest model (RFM) and partial least squares path model (PLS-PM) demonstrated that OMM increased faba bean productivity by improving soil microbial diversity and the efficiency of C cycling.

**Conclusion:**

This study highlighted that OMM could promote C and N cycling by regulating core microbial groups, thereby improving the faba bean productivity in dryland of purple soil hilly region of southwest China.

## 1 Introduction

Agroindustrial waste such as crop straw and legume green manure are high-quality organic mulch materials, which play an important role in improving soil ecological environment, soil quality, and crop yield (Chen S. H. et al., 2022). Crop straw to corn (*Zea mays* L.), faba bean (*Vicia faba* L.) and rape (*Brassica campestris* L.) is much abundant as a resource of renewable cellulose in farmland resources and yet it is underutilized (Yu S. et al., 2022). Straw is rich in cellulose, hemicellulose, lignin, and ash, as well as a rich source of carbon (C) (Wang et al., 2021). According to the agricultural production statistics of the Food and Agriculture Organization (FAO) (FAOSTAT, 2021) and the crop straw index (Zhang et al., 2021), the total global straw output in 2020 was about 5.849 billion tons, with China accounting for 17.85% which is the largest straw producer in the world. In 2021, the return rate of crop straw in China was about 61.82%, and organic material mulching (OMM) holds immense potential for scientific research (Yang et al., 2022). Since crop straws are characterized by a component of high carbon (C) to nitrogen (N) ratio (C:N), their return to the field leads to a competition between soil microorganisms and crops for mineral N, resulting in high greenhouse gas emissions (Hassan et al., 2024; Xin et al., 2024). Further, it can lead to a significant imbalance in the soil microbial community structure (Xie et al., 2022b). Therefore, determining the approach to optimize the interactions among soil microbes, soil quality, and crops after crop straw return is a crucial prerequisite to ensure the scientific utilization of straw resources. Milk vetch (*Astragalus sinicus* L.) is a high-quality legume green fertilizer, which can contribute to supplementing the nutrient requirements of cultivated land and replace part of fertilizer usage upon its return to the field (Ma et al., 2020). Moreover, it enriches the diversity of microbial community structure (Xia et al., 2023). The most important benefit of milk vetch is that it can reduce soil C:N (Fang et al., 2024), which helps in alleviating the increase in C:N after the return of straw to the field, and also promotes the release of mineral N by microbial decomposition of organic materials (Xie et al., 2022a). However, the mineralization of straw and milk vetch is a relatively slow process in the soil (Xu et al., 2021). Currently, there are limited long-term dynamic studies to understand the effects of mulching different crop straws in combination with milk vetch on soil microorganisms and soil quality.

Purple soil region of southwest China is a mountainous and hilly area, with high temperatures, high humidity, low levels of sunlight, and regular human activities (Tan et al., 2021), which has resulted in a fragile ecological environment and lack of cultivated land resources (Chu et al., 2020). At the same time, the extensive area under spring and summer grain crops in purple soil hilly region of southwestern China and the fallow time after harvest leads to a low multiple cropping index, resulting in a significant waste of natural resources (Chu et al., 2020). The multi-cropping system, commonly practiced in the purple soil hilly region of southwest China (Wu et al., 2023), effectively utilizes light, heat, water, and limited cultivated land resources to enhance farmland ecosystem diversity and ensure food production (He et al., 2023; Nasar et al., 2023). However, a multi-cropping system requires large amounts of nutrients for land cultivation. In addition, complex issues such as a wide range of crop options, greater niche competition, unreasonable fertilization, and extensive tillage practices are associated with this system (Chen et al., 2022). The selection of core crops based on the local conditions is vital for optimizing the soil microecological environment and improving soil quality. Faba bean is an important crop in the multi-cropping system in purple soil hilly region of southwest China, where the area under faba bean production is the largest in China (Zhou et al., 2022). The cultivation of faba bean provides multiple benefits such as improvement in the multiple cropping index, leguminous crop-based N fixation, enhancing the growth of numerous beneficial rhizosphere microorganisms, and stabilizing the soil ecosystem (Karkanis et al., 2018). More importantly, the faba bean as the major crop of the multi-cropping system can improve the soil quality of the fragile agricultural land and enhance the cropping index in purple soil hilly region of southwest China (Jithesh et al., 2024; Nasar et al., 2024). On the other hand, the multi-cropping system has significant advantages such as the production of straws, which are rich sources for OMM. Therefore, it is essential to investigate the multi-cropping system with the faba bean as the core crop for improving the soil ecosystem and stabilizing grain production in the purple soil hilly region of southwest China.

Soil microorganisms are key biological factors driving biotic and abiotic processes in farmland ecosystems (Barq et al., 2021). They play a vital role in soil N cycling (Zhang et al., 2022), contribute to collaborative crop resilience processes (Grobelak et al., 2018), and maintain ecosystem stability (Bhaduri et al., 2022). Straw mulching can regulate the changes in soil microbial community structure and function (Xu et al., 2023). The variations in organic materials composition and C:N ratio of different crop straws is the major reason for the changes in the soil microecological environment (Chen L.M. et al., 2022). Su et al. (2020) demonstrated the presence of copiotrophic bacteria mainly in the soil under corn straw return, while oligotrophic bacteria were mainly found in the soil under wheat straw return. Mulching and returning the crop straw to the field can enhance microorganisms that play key roles in N fixation, nitrification, and denitrification (Dou et al., 2023), stimulate the activity of sulfur-reducing bacteria, and reduce soil acidification (Zhong et al., 2021). In particular, milk vetch can effectively improve the N-use efficiency of soil microorganisms and maintain a suitable C:N ratio under crop straw mulching (Li et al., 2021). Gao et al. (2021) found that the return of milk vetch to rice soil had a significant impact on the community and function of soil bacteria, increasing the abundance of *Actinobacteria* and *Firmicutes*, and also enhancing nutrient cycling. All studies mentioned above indicated that the impacts of diverse organic materials inputs on soil microbial community structure and function are complex. Further research on the ecological benefits provided by the soil microorganisms under the multi-cropping system with different organic mulching is of great significance for the sustainable development of agricultural production in purple soil hilly region of southwest China.

According to previous studies, the ecological mechanism that explains the role of OMM in dynamically driving the soil nutrient cycling and changes in the microbial community structure that affect faba bean yield in the context of the triple cropping model in purple soil hilly region of southwest China is still unclear. Therefore, in this study, a field experiment was designed for two consecutive years in typical purple soil farmland in Chongqing, China. It was assumed that mulching straw combined with milk vetch onto the field has the potential to gradually improve the physicochemical properties and the diversity of microbial community in the soil, which leads to an increase in faba bean productivity. The objectives of this study were (1) to investigate the dynamic effects of straw and milk vetch mulching on the soil physicochemical properties and faba bean productivity; (2) to explore the dynamic changes in the stability of core microbial functions and molecular ecological networks (MENs) in faba bean soil following straw and milk vetch mulching; (3) to elucidate the ecological mechanism of increase in faba bean productivity associated with the changes in soil physicochemical properties and microbial communities driven by straw and milk vetch mulching. Briefly, this study under conservation tillage with OMM can provide a theoretical basis and practical guidance for the multi-cropping system in purple soil hilly region of southwest China to alleviate farmland soil erosion, improve ecological benefits, and stabilize sustainable grain production.

## 2 Materials and methods

### 2.1 Site description and experimental design

**Figure 1:**
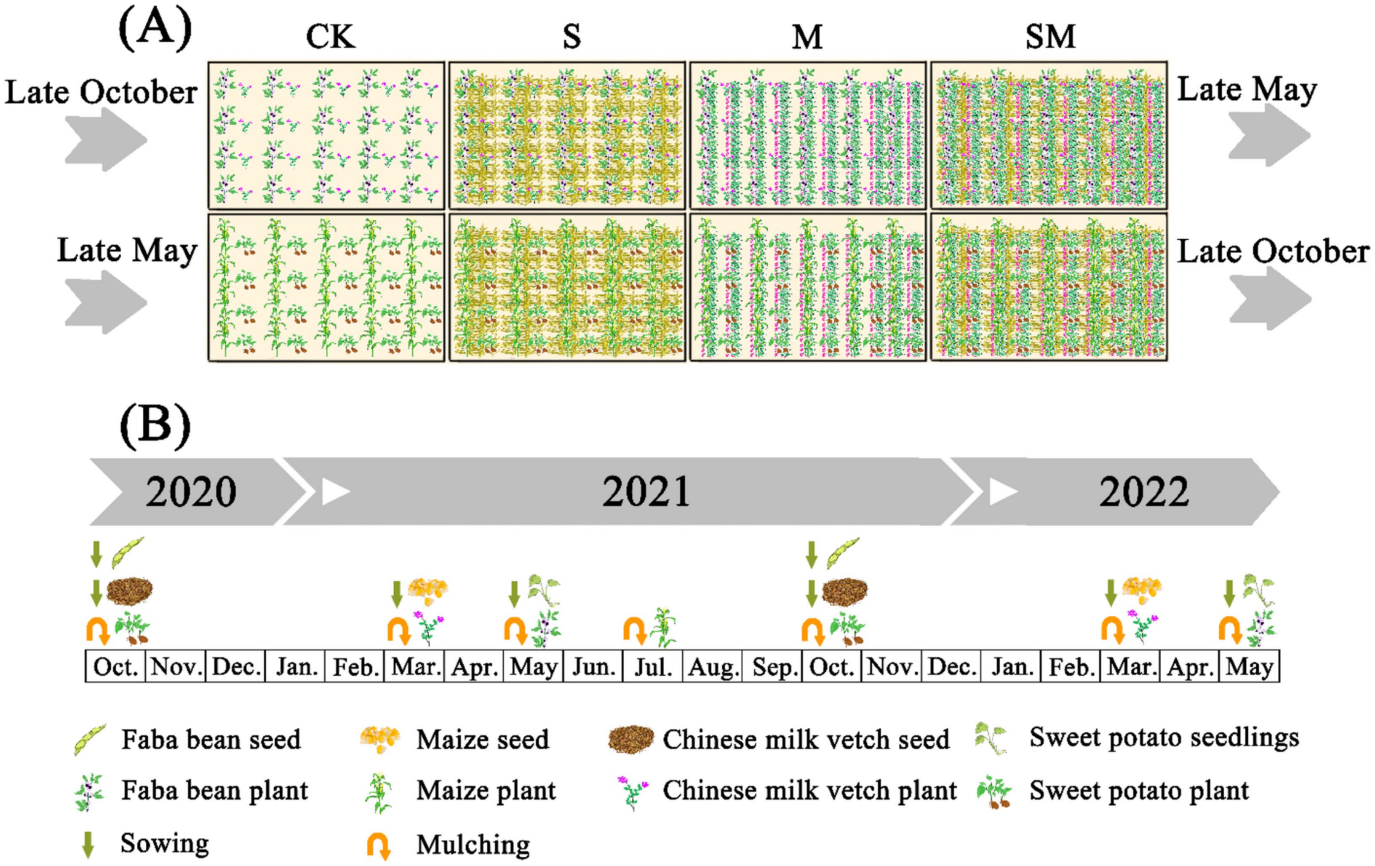
Mulching with organic materials **(A)** and faba bean-corn-sweet potato three-ripe preparation succession scheme **(B)**. CK, without organic material mulching; S, straw mulching; M, milk vetch mulching; SM, straw and milk vetch mulching.

The experiment was conducted in the National Purple Soil Fertility and Fertilizer Benefit Monitoring Base (29°51′N, 106°27′E, and altitude 244 m) of Southwest University, Beibei District, Chongqing, China from October 2020 to May 2022. The experimental site has a subtropical humid climate with an average annual temperature of 18.3°C and an average annual rainfall of 1,105 mm. The soil type is purple soil, which is derived from the taupe purple sandy mudstone of the Jurassic Shaximiao Formation (J2s) (Zuo et al., 2020). The physicochemical properties of the soil in the top layer (0–15 cm) were pH—6.54, bulk density—1.1 g·cm^−3^, soil organic carbon (SOC)—13.50 g·kg^−1^, total nitrogen (TN)—1.18 g·kg^−1^, total phosphorus (TP)—0.9 g·kg^−1^, total potassium (TK)—20.38 g·kg^−1^, alkaline hydrolyzable nitrogen (AN)—101.68 g·kg^−1^, available phosphorus (AP)—74.98 g·kg^−1^, and available potassium (AK)—243.13 g·kg^−1^. The slope of the test field is shallow and the soil fertility is relatively uniform. This study was based on the faba bean-corn-sweet potato intercropping model in the multi-cropping system in dry land. In this intercropping model, the faba bean variety is the major local variety of small green faba bean, the corn variety is Zhengda 999, and the sweet potato (*Ipomoea batatas* Lam.) variety is Yuhongxin 98. The experiment was characterized by a single-factor randomized block design, and a total of four treatments was set up, namely, without organic material mulching (CK), straw mulching (S), milk vetch (*Astragalus sinicus* L.) mulching (M), and straw and milk vetch mulching (SM) (Supplementary Table S1). The treatments were replicated for three times, and each plot was 8 m long, 4 m wide, and 32 m^2^ in area. The crop establishment and management plans are presented in Figure 1A.

Faba bean was sown in late October each year, with two rows planted in each strip (row spacing of 30 cm, hole spacing of 30 cm, and hole depth of 5 cm). Subsequently, the seedlings were thinned and patched after emergence and harvested in early May of the following year. Corn was sown in late March each year, with two rows planted in each strip (row spacing 65 cm, plant spacing 30 cm, and seeding depth 15 cm), and harvested in late July. Sweet potato seedlings were transplanted using the direct insertion method in late May each year, with three rows planted in each strip (row spacing of 65 cm, hole spacing of 25 cm, 16 holes in each row, and 2 plants in each hole depth of 10 cm), and harvested in late October. Milk vetch was sown in uniform lines with bare seeds at a rate of 75 kg·ha^−1^, and intercropped with faba beans. The seeds were planted in late October each year, and the waxy layer on the surface of the seed coat was removed before sowing. The crop planting sequence and returning operation are presented in Figure 1B.

During the growth phase of each crop, compound fertilizer (N:P_2_O_5_:K_2_O = 16:16:16) was applied once as the base fertilizer at a rate of 200 kg·ha^−1^ and also applied to the holes at the time of sowing or transplantation. In late March every year, urea at a rate of 115 kg·ha^−1^ was applied as the flower and pod fertilizer for faba beans, and 0.2% potassium dihydrogen phosphate solution was sprayed on the leaf surface 2–3 times. In early May, 270 kg·ha^−1^ of urea was applied to corn plantation during the jointing stage and 150 kg·ha^−1^ of urea was applied to sweet potato plantation during the growth phase. Other field management practices applicable for each crop are the same as local conventional cultivation methods. Fresh samples of straw were randomly collected after crop harvesting in the current season and also during the blooming period of milk vetch. The samples were initially oven-dried at 105°C for 30 min and then further dried to constant weight at 65°C. After grinding, the nutrient contents of each sample of crop straw and milk vetch were analyzed (Supplementary Table S2).

### 2.2 Plant and soil sampling and analysis

Field soil samples were collected from 0 to 15 cm top layer using the point-centered quarter sampling method with a soil drill from May 1 to 3 in 2021 and 2022. According to the point-centered quarter sampling method, 1.5 kg of samples were collected and stored in a sealed bag. All the samples were immediately transported to the laboratory and kept at 4°C. The collected soil samples were screened using a 2 mm sieve to remove plant residues, fine roots, stones, and other debris. Subsequently, each sample was divided into three parts. One part of the fresh soil sample was stored at −80°C for examination of soil microorganisms. The other part of the fresh soil sample was stored at 4°C for the determination of soil enzyme activity. The remaining part of the soil sample was air-dried for soil aggregate measurement and chemical characterization.

### 2.3 Soil aggregate fractionation

The primary methods for evaluating the stability of soil aggregates are the wet-dry sieve method. The dry sieving method assesses the mechanical stability of aggregates, while the wet sieving method evaluates their water stability.

*Separation of dry aggregates*: the aggregate samples underwent size classification through dry sieving using an aggregate sieving apparatus (TPF-100, Shanghai Tomos Scientific Instrument Co., Ltd.). Approximately 200 g of soil samples were processed through a trio of sieves featuring different mesh sizes to obtain four distinct aggregate size fractions. The particles experienced oscillation for 10 min at a frequency of 200 cycles per minute with an amplitude of 2 mm, followed by weighing each particle size. The soil collected on the 2,000 μm, 250 μm, and 106 μm sieves was carefully gathered and categorized as large macro-aggregates (>2,000 μm), small macro-aggregates (250–2,000 μm), micro-aggregates (106–250 μm), and silt and clay particles (<106 μm), respectively (Wang et al., 2016).

*Separation of wet aggregates*: A mixed soil sample weighing 50 g was prepared according to the proportions of soil particle sizes determined by the dry sieving technique and placed on top of the sieves (dimensions: 2,000 μm, 250 μm, 106 μm). Deionized water was used to reach the water level, ensuring that it sufficiently submerged the sieves. The setup was then allowed to stand in the bucket for 10 min. Following this, the samples underwent oscillation at a frequency of 30 times per minute with an amplitude of 2 mm for a duration of 10 min. The aggregates from each sieve were subsequently collected by rinsing with deionized water and were then moved into aluminum boxes for drying in a thermostatically controlled electric oven set at 105°C.

To determine the distribution of soil aggregates, the proportion of each fraction based on dry weight was calculated. Additionally, metrics such as macro-aggregate content (*R*_0.25_), percentage of aggregate destruction (PAD), mean weight diameter (MWD), geometric mean diameter (GMD), and fractal dimensions (Ds) were derived as indicators of aggregate stability, following the Equations 1–5 outlined by Tagar et al. (2020) and Zhu et al. (2020).


(1) $$R_{0.25}=DR_{0.25}/{\mathrm{DM}}_{T}\times 100\%$$

(2) $$\mathrm{PAD}=(DR_{0.25}-WR_{0.25})/DR_{0.25}\times 100\%$$

(3) $$\mathrm{MWD}=\sum_{i=1}^{n}X_{i}\times M_{i}$$

(4) $$\mathrm{GMD}=\exp[\frac{\sum_{i=1}^{n}M_{i}\times \mathrm{Ln}X_{i}}{\sum_{i=1}^{n}M_{i}}]$$

(5) $$\log_{10}\{\frac{M(\delta <\bar{X_{i}})}{M_{0}}\}=(3-\mathrm{Ds})\log_{10}\frac{\bar{X_{i}}}{X_{\max}\;}$$

Where, $DR_{0.25}$ is the greater than 0.25 mm (250 μm) mechanical-stable aggregates mass. ${\mathrm{DM}}_{T}$ is the mechanical-stable aggregate mass. $WR_{0.25}$ is the greater than 0.25 mm (250 μm) water-stable aggregates mass. *X_i_* is the mean diameter (mm) of size *i* aggregates in any aggregate size fraction, and *M_i_* is the weight of size *i* aggregates in that aggregate size fraction as a proportion of the dry weight of soil. $M(\delta <\bar{X_{i}})$ is the cumulative soil aggregates mass (g) less than the average of two adjacent aggregate sizes, *M*_0_ is the sum of the mass of soil aggregates of each aggregate size (g), $\bar{X_{i}}$ is the mean particle diameter (mm) of size *i* aggregates and is considered as the arithmetic mean of the upper and smaller sieve sizes, *X_max_* is the mean diameter of the aggregate with the maximum size, and *Ds* is the fractal dimension of soil aggregates (dimensionless).

### 2.4 Chemical analyses

Soil water content (SWC) was determined from mass loss after drying at 105°C for 24 h following the method by Zhao et al. (2023). The chemical properties of soil were measured as described by Bao (1999). Soil pH was measured by potentiometric method (soil: water ratio of 1:5), soil organic carbon (SOC) was determined by potassium dichromate capacity method, soil total nitrogen (TN) was determined by micro-Kjeldahl method, soil total phosphorus (TP) was determined by H_2_SO_4_–HClO_4_ digestion and molybdenum-antimony anti-colorimetric method, and soil total potassium (TK) was NaOH fusion-flame photometry.

Soil hydrolytic nitrogen (AN) was determined by alkaline diffusion method, and soil nitrate nitrogen (NO_3_^−^–N) and ammonium nitrogen (NH_4_^+^–N) were determined by continuous flow analyzer method (Zhang et al., 2015). Soil available phosphorus (AP) was determined by sodium bicarbonate extraction-molybdenum-antimony resistance colorimetric method (Ahmed et al., 2020). Soil available potassium (AK) was determined by ammonium acetate extraction-flame photometer (Lu et al., 2017).

### 2.5 Soil enzyme assays

Extracellular enzyme activities were determined using an enzyme-linked immunosorbent assay (ELISA) kit (Jiangsu Meimian Industrial Co., Ltd., Yancheng, China) following the manufacturer’s instructions. Activities of six hydrolytic soil enzymes were measured, including two enzymes involved in C-acquisition: β-1,4-glucosidase, β-1,4-xylosidase; two enzymes involved in N-acquisition: urease, L-leucine aminopeptide; and one enzyme involved in P-acquisition: acid phosphatase. In addition, we measured activities of one oxidase involved in the degradation of recalcitrant organic C: peroxidase (Tian et al., 2022). All the above activities were represented by μmol·g^−1^·h^−1^ dry soil. To represent the general potential C, N, and P-acquiring enzyme activity, the C, N, and P acquiring enzymes were grouped and normalized as ln (β-1,4-glucosidase + β-1,4-xylosidase), ln (Urease + L-leucine aminopeptide), and ln (acid phosphatase), respectively (Sinsabaugh et al., 2009).

### 2.6 Extracting DNA from soil, Illumina MiSeq sequencing, and microbial co-occurrence network construction

Sample preparation and DNA extraction consist of the following steps: Soil specimens were flash-frozen in liquid nitrogen and stored at −80°C to prevent nucleic acid degradation. Total genome DNA from soil samples was extracted by the CTAB method (Kamdem et al., 2023). DNA concentration and purity were monitored on 1% (w/v) agarose gels. According to the concentration, DNA was diluted to l μg·μL^−1^ using sterile water. 16S rRNA/18S rRNA/ITS genes of distinct regions (16S V4/16S V3/16S V3-V4/16S V4-V5, 18S V4/18S V9, ITS1/ITS2, Arc V4) were amplified by specific primer 16S rRNA (515F: GTGCCAGCMGCCGCGGTAA and 907R: CCGTCAATTCCTTTGAGTTT) and ITS (ITS1-1F-F: CTTGGTCATTTAGAGGAAGTAA and ITS1-1F-R: GCTGCGTTCTTCATCGATGC) with the barcode. All PCR reactions were carried out with 15 μL of Phusion® High-Fidelity PCR Master Mix (New England Biolabs), 0.2 μM of forward and reverse primers, and about 10 ng template DNA. Thermal cycling consisted of initial denaturation at 98°C for 1 min, followed by 30 cycles of denaturation at 98°C for 10 s, annealing at 50°C for 30 s, and elongation at 72°C for 30 s, finally extension at 72°C for 5 min. Reactions were performed in triplicate under optimized cycling conditions (30–35 cycles) to minimize amplification bias. Amplicons were purified using AMPure XP beads and quantified via Agilent Bioanalyzer. The library was constructed using the TruSeq® DNA PCR-Free Sample Preparation Kit. The constructed libraries were quantified using Qubit and quantitative PCR (Q-PCR). Once the libraries were validated, they were sequenced on the NovaSeq 6000 platform. Finally, the libraries underwent sequencing on the Illumina NovaSeq platform. The raw sequence data reported in this paper have been deposited in the Genome Sequence Archive in the National Genomics Data Center, Beijing Institute of Genomics (China National Center for Bioinformation), Chinese Academy of Sciences, under accession project numbers CRA011413 and CRA011419 for 16S rRNA and ITS rRNA genes, respectively, which are publicly accessible at https://bigd.big.ac.cn/gsa (Edgar, 2004; Edgar, 2013; Quast et al., 2013). More details can be found in Appendix A. Supplementary material (Guimerà and Amaral, 2005; Tolosana-Delgado, 2008; Zhou et al., 2011; Deng et al., 2012; Luo et al., 2014; Ju and Zhang, 2015; Wu et al., 2021; Mao et al., 2022).

### 2.7 Grain yield and the economic value of faba bean

The yield of faba bean were measured at the maturity stage. The faba bean seeds were collected separately from each plot, and the weight of the seeds after the dry pod threshing was measured and recorded as the grain yield of the corresponding plot. The economic value was calculated based on the current year’s price of faba beans.

### 2.8 Data analysis and statistics

The data obtained in this study were maintained in Microsoft Excel 2016. Statistical analyses were performed using R software (Version 4.2.1). Data were subjected to Levene’s test for homogeneity of variance and the Shapiro–Wilk test for normality. One-way ANOVA analysis was performed to compare the effects of soil physicochemical properties, soil biological characteristics, and economic properties among different treatments (*p* = 0.05). Qiime software (Version 1.9.1) was employed to calculate Chao1, Shannon, Simpson, and Ace indices. The differences in the alpha diversity index between groups were further analyzed using R software. Two-way ANOVA and Bonferroni test were performed to examine the significant differences in soil aggregate stability, chemical composition, enzyme activity, and microbial diversity across different years and among diverse treatments (*p* = 0.05). Further details of data analyses can be found in Appendix A of Supplementary material.

## 3 Results

### 3.1 Impacts of organic mulch materials on the soil physicochemical properties and enzymatic activities

**Figure 2:**
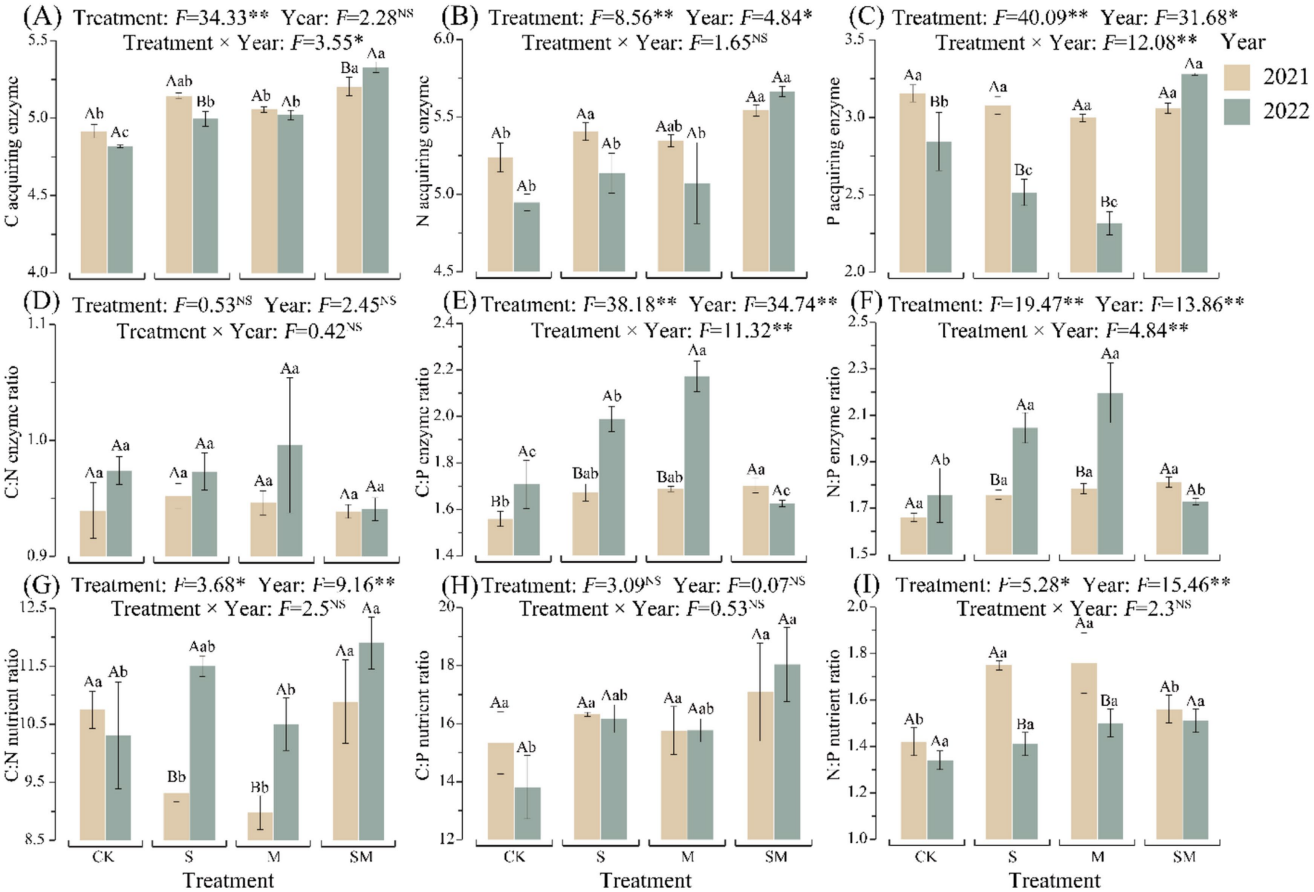
Enzyme activities, enzymatic stoichiometry, and soil nutrient stoichiometry under treatments using different organic mulching materials. **(A)** Activities of normalized C-acquiring enzymes. **(B)** Activities of normalized N-acquiring enzymes. **(C)** Activities of normalized P-acquiring enzymes. **(D)** C:N acquiring enzyme activity ratio. **(E)** C:P acquiring enzyme activity ratio. **(F)** N:P acquiring enzyme activity ratio. **(G)** C:N ratio. **(H)** C:P ratio. **(I)** N:P ratio. Different lowercase letters indicate significant differences between different treatments of the same year. Different capital letters indicate significant differences between different years of the same treatment. CK, without organic material mulching; S, straw mulching; M, milk vetch mulching; SM, straw and milk vetch mulching.

OMM treatment (S, M, and SM), as well as the year of plantation and their interactions, had significant effects on soil aggregate stability, nutrients content, enzymatic activity, and enzyme stoichiometric ratios (*p* < 0.05) (Supplementary Tables S3, S4; Figure 2 and Supplementary Figure S1). In 2022, compared to CK, soil aggregate stability of OMM treatment significantly improved (*p* < 0.05). In 2021 and 2022, compared to CK, the soil organic carbon (SOC), total nitrogen (TN) content, and the activities of soil C and N acquiring enzymes of OMM treatment increased. Soil macro-aggregate content (*R*_0.25_) and SOC increased with the increase in mulching duration. All the results mentioned above indicate that the parameters increased with SM treatment compared to other OMM treatments.

### 3.2 Effects of organic mulching materials on soil microbial community structure

**Figure 3:**
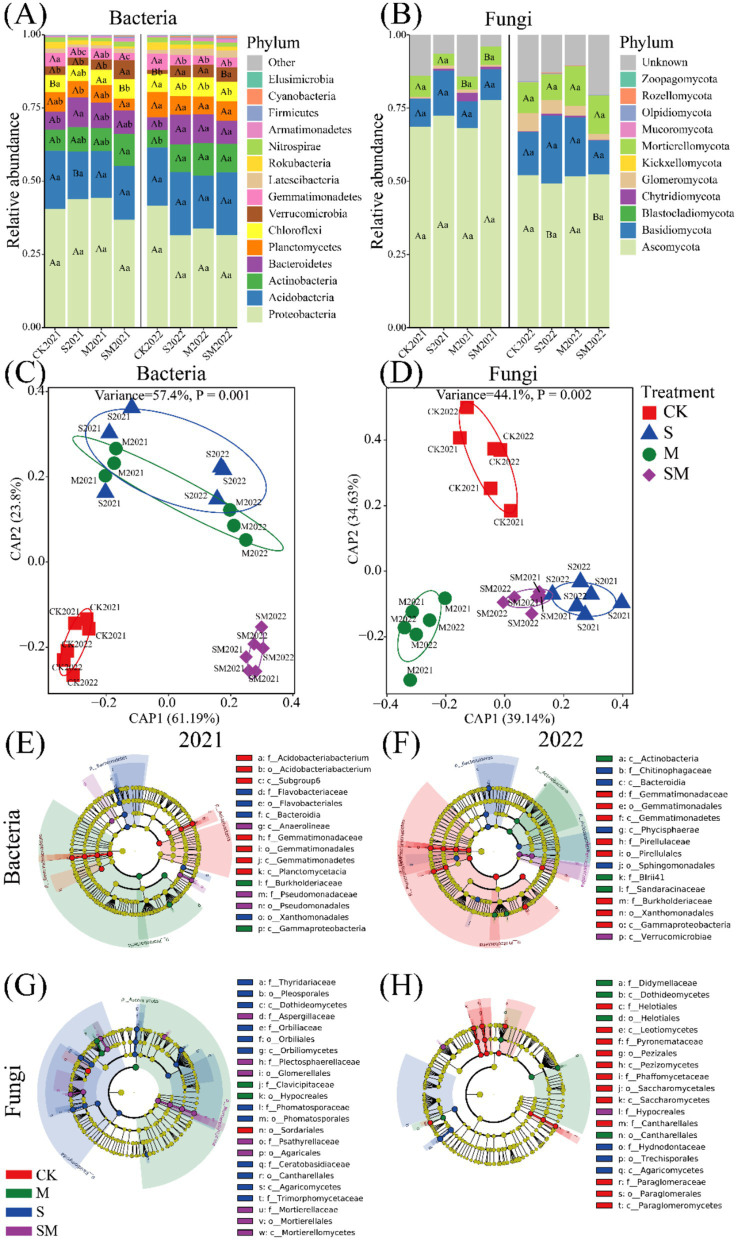
Differences in the microbial communities between CK and organic mulch treatments. Relative abundance of soil bacteria **(A)** and fungi **(B)** at the phylum level, CPCoA of soil bacteria **(C)** and fungi **(D)**. Biomarkers of cultivated ginseng soil microbiota at different organic mulch treatments using the LEfSe method. Only results with |LDA| > 4 and *p* < 0.05 according to Tukey’s honest significant difference test are shown. **(E,F)** Bacterial biomarkers in the soil samples obtained from fields in 2021 and 2022, respectively. **(G,H)** Fungal biomarkers in the soil samples obtained from 2021 and 2022, respectively. Different lowercase letters indicate significant differences between different treatments of the same year. Different capital letters indicate significant differences between different years of the same treatment. CK, without organic material mulching; S, straw mulching; M, milk vetch mulching; SM, straw and milk vetch mulching.

In 2021 and 2022, the relative abundance of the dominant bacterial communities at the phylum level, i.e., *Proteobacteria* and *Acidobacteria*, among treatments demonstrated no significant difference (Figure 3A). However, the relative abundance of *Actinobacteria* and *Acidobacteria* in OMM treatment soils was higher than that in CK, i.e., an increase of 66.67 and 150%, respectively in 2022 (*p* < 0.05). Further, in 2021 and 2022, the relative abundance of dominant fungal communities at the phylum level, i.e., *Ascomycota*, *Basidiomycota*, *Glomeromycota*, and *Mortierellomycota* among treatments demonstrated no significant difference (Figure 3B).

The results of bacterial α-diversity indicated that in 2021, the observed species, Shannon index, and Simpson index in the SM treatment were significantly higher than those in the other treatments (Supplementary Table S5). In 2022, the observed species and Shannon indices in OMM treatment soils significantly increased by 7.7–18.85% and 6.05–8.48%, respectively (*p* < 0.05), when compared with CK. Moreover, the bacterial α diversity indices increased with the increase in mulching time. The fungal α diversity results demonstrated no significant difference among treatments in 2021 (Supplementary Table S6). However, in 2022, except for the Shannon index, other fungal α diversity indices in OMM treatment soils were higher than those in CK. The above results indicated greater improvement in the SM treatment when compared with other OMM treatments. The constrained principal coordinate analysis (CPCoA) of β diversity results for 2021 and 2022 (Figures 3C,D) demonstrated significant differences in the distribution of bacterial and fungal communities between CK and OMM treatments (*p* < 0.05), which corresponded to 57.4 and 44.1% of the total changes in the community, respectively.

The results of this study indicate that the predominant bacterial groups at the phylum level in CK were *Gemmatimonadetes*, *Acidobacteria*, and *Planctomycete*; and at the class level were *Gemmatimonadetes*, *Subgroup 6*, and *Planctomycetacia* (Figures 3E,F; Supplementary Figure S2A; Supplementary Table S9). The predominant bacterial groups at the phylum level in OMM treatment soil were *Proteobacteria*, *Actinobacteria*, and *Bacteroidetes*; and at the class level were *Gammaproteobacteria*, *Actinobacteria*, and *Bacteroidia*. In 2021 and 2022, the predominant fungal groups at the phylum level in CK were *Ascomycota* and *Glomeromycota*, and at the class level were *Paraglomeromycetes* and *Saccharomycetes*. Similarly, the predominant fungal groups in OMM treatment soil at the phylum level were *Ascomycota* and *Basidiomycota*, and at the class level were *Sordariomycetes*, *Dothideomycetes*, and *Agaricomycetes*. In addition, the major bacteria in the SM treatment soil at the class level were *Anaerolineae* and *Verrucomicrobiae*, and the major fungus in SM treatment soil at the class level was *Mortierellomycetes* (Figures 3G,H; Supplementary Figure S2B; Supplementary Table S9).

### 3.3 Effects of organic mulching materials on molecular ecological network characteristics of soil microbial communities

**Figure 4:**
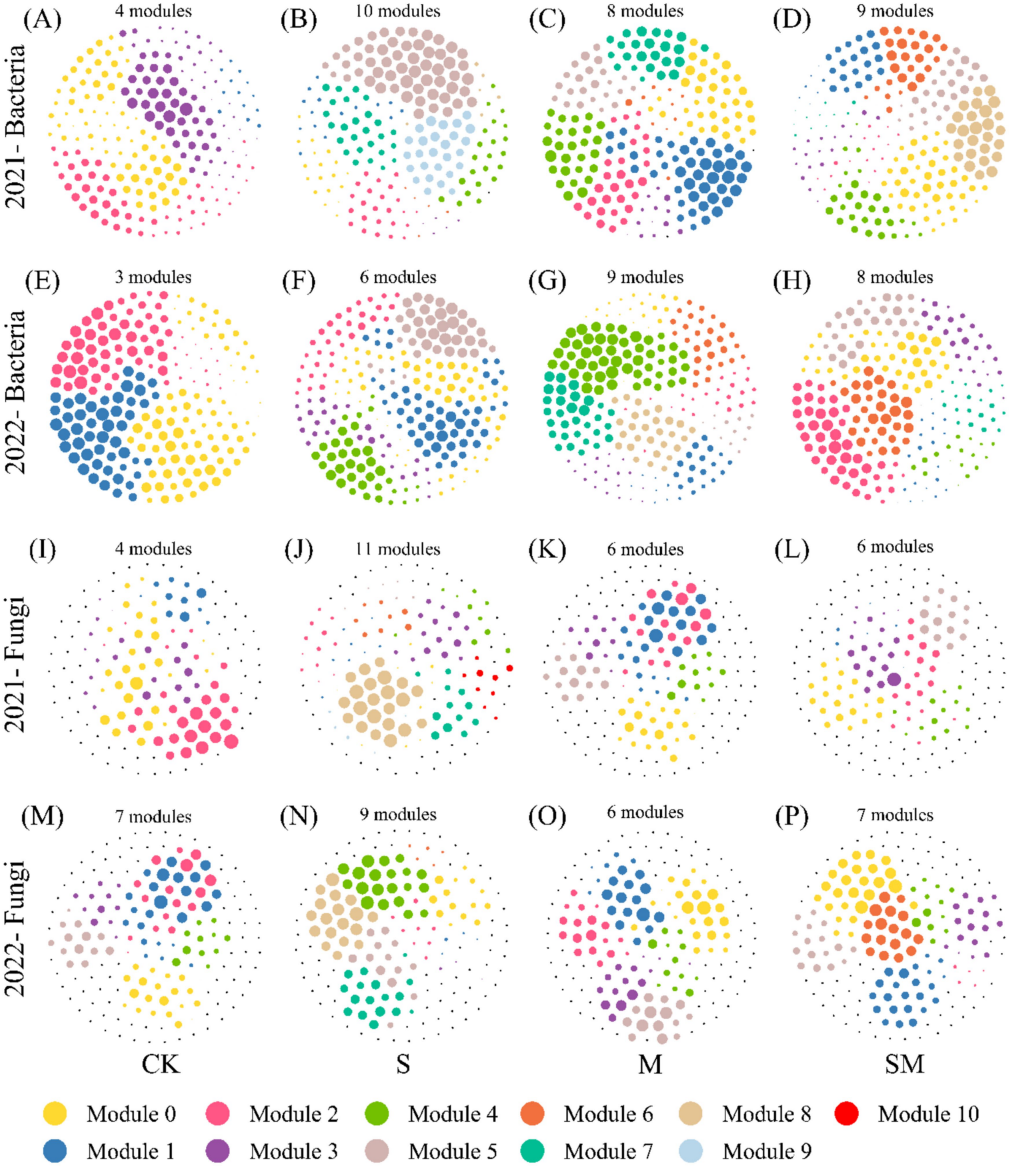
Co-occurrence networks of soil bacteria and fungi. **(A–D)** Bacteria in CK and S, M, and SM treatment fields in 2021. **(E–H)** Bacteria in CK and S, M, and SM treatment fields in 2022. **(I–L)** Fungi in CK and S, M, and SM treatment fields in 2021. **(M–P)** Fungi in CK and S, M, and SM treatment fields in 2022. CK, without organic material mulching; S, straw mulching; M, milk vetch mulching; SM, straw and milk vetch mulching.

A comparative analysis of the molecular ecological network between CK and OMM treatments revealed improvements in the size and complexity of the microbial network under OMM treatments, particularly in terms of total nodes, total links, average degree, and total modularity number. In addition, the microbial molecular ecological network characteristics treated by OMM in 2022 were superior to those in 2021 (Figure 4; Supplementary Tables S7, S8).

The key soil microbial species in CK and OMM treatments were found in the connectors and module hubs of the MENs (Supplementary Figure S3; Supplementary Table S12) of soil microbial communities. In 2021 and 2022, the key bacterial species in CK at the phylum level were *Gemmatimonadetes*, *Proteobacteria*, and *Acidobacteria,* and at the class level were *Gemmatimonadetes*, *Alphaproteobacteria*, and *Acidobacteriia*. The key bacterial species in OMM treatment soils at the phylum level were *Proteobacteria* and *Acidobacteria* and at the class level were *Alphaproteobacteria*, *Gammaproteobacteria*, and *Acidobacteriia*. Further, in 2021 and 2022, the key fungus in CK at the phylum level was *Ascomycota,* and at the class level was *Sordariomycetes*. The key fungi in the OMM treatment soils at the phylum level were *Ascomycota* and *Basidiomycota*, and at the class level were *Sordariomycetes*, *Eurotiomycetes*, and *Agaricomycetes.* Specifically, the fungus in SM treatment at the class level was *Glomeromycetes*.

### 3.4 Relationship among soil environmental factors, microbial community structure, and soil microbial predicted functions

**Figure 5:**
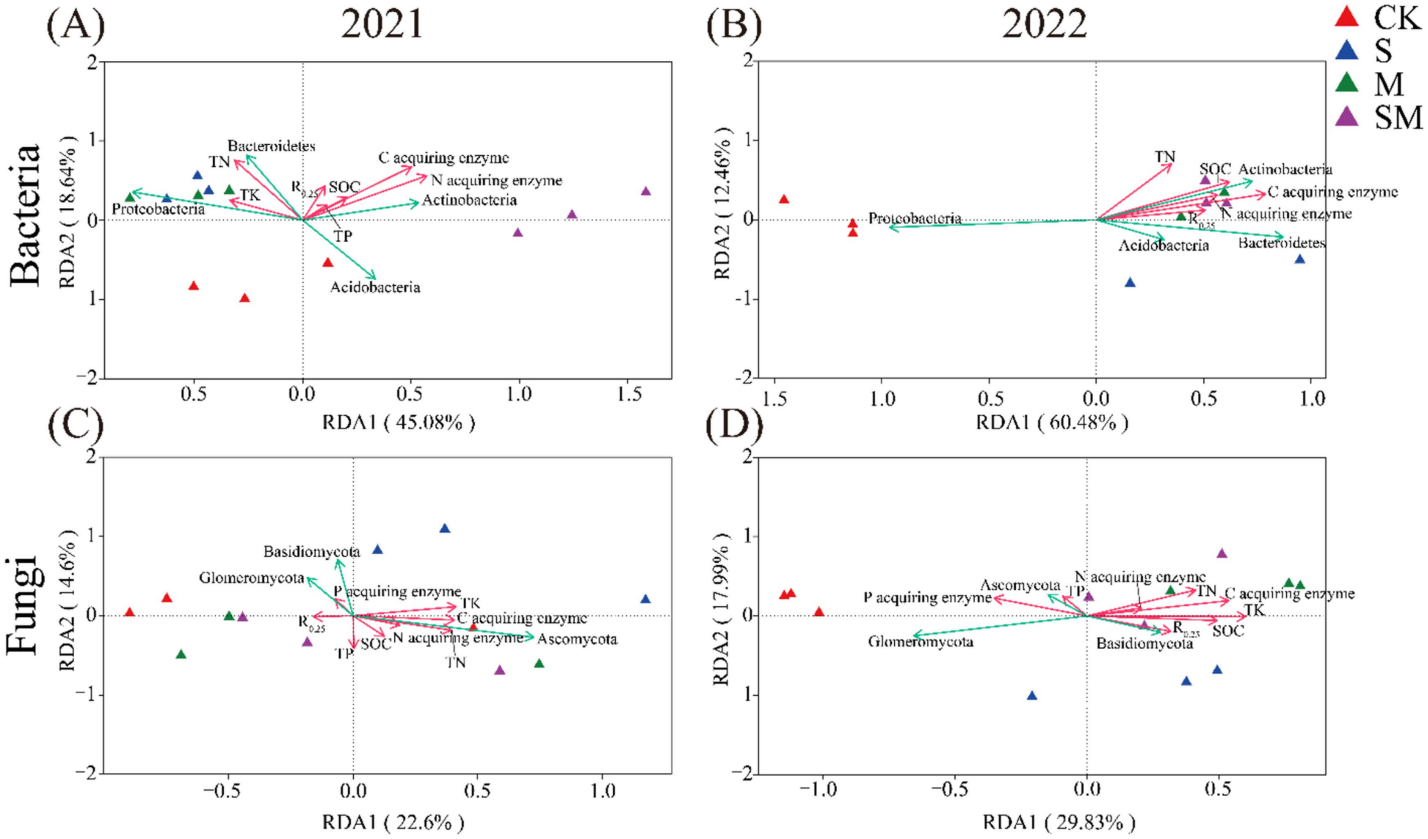
Redundancy analysis (RDA) of the soil microbial community structure and physicochemical factors in the fields. **(A)** Bacteria in 2021, **(B)** Bacteria in 2022, **(C)** Fungi in 2021, **(D)** Fungi in 2022. CK, without organic material mulching; S, straw mulching; M, milk vetch mulching; SM, straw and milk vetch mulching.

A significant linear relationship was observed between environmental factors and bacterial community structure in 2021 (*p* = 0.002; Supplementary Table S15). The redundancy analysis (RDA) of the bacterial community structure revealed that environmental factors accounted for 63.72% of the total variance. The C-acquiring enzyme activity (variation accounting for 15.48%, *p* = 0.002) and N-acquiring enzyme activity (variation accounting for 13.62%, *p* = 0.001) were the major environmental factors influencing the bacterial communities. Both these factors had a positive correlation with *Actinobacteria*. Additionally, the SM had a strong positive correlation with C-acquiring enzyme activity, N-acquiring enzyme activity, and *Actinobacteria* (Figure 5A). In 2022, the RDA of the bacterial community structure revealed that the environmental factors accounted for 72.94% of the total variance. C-acquiring enzyme activity (variation accounting for 32.9%, *p* = 0.121) and SOC content (variation accounting for 14.9%, *p* = 0.311) were the major environmental factors influencing the bacterial communities. These factors were positively correlated with *Actinobacteria*, *Acidobacteria*, and *Bacteroidetes*. OMM had a strong positive correlation with C acquiring enzyme activity, SOC content, *Actinobacteria*, *Acidobacteria*, and *Bacteroidetes* (Figure 5B).

In 2021 and 2022, a significant linear relationship was observed between the environmental factors and fungal community structure. The RDA of the fungal community structure demonstrated that the environmental factors accounted for 37.2% of the total variance in 2021. The C-acquiring enzyme activity (variation accounting for 10%, *p* = 0.059) and TN content (variation accounting for 10.7%, *p* = 0.085) were the major environmental factors influencing the fungal communities. Both these factors were positively correlated with *Ascomycota* (Figure 5C). In 2022, the RDA of the fungal community structure demonstrated that the environmental factors accounted for 47.82% of the total variance. Among them, TK content and C-acquiring enzyme activity were the major environmental factors influencing the fungal communities and were positively correlated with *Basidiomycota*. OMM had a strong positive correlation with TK content, C acquiring enzyme activity, SOC content, TN content, and *Basidiomycota* (Figure 5D).

In 2021 and 2022, when compared with CK, PICRUSt predicted that OMM significantly up-regulated 5 first-order biological metabolic pathways (Level 1) and 17 s-order biological metabolic pathways (Level 2) (*p* < 0.05). The 5 Level 1 pathways included genetic information processing (Level 2: genetic information processing unclassified, folding, sorting, degradation, translation and replication, and repair), metabolism (Level 2: glycan biosynthesis and metabolism, enzyme families, and energy metabolism), cellular processes (Level 2: cell motility and cell communication), environmental information processing (Level 2: signal transduction, metabolism of cofactors and vitamins, and nucleotide metabolism), and organismal systems (Level 2: digestive system, immune system, environmental adaptation, and sensory system) (*p* < 0.05) (Supplementary Table S13).

In 2021 and 2022, when compared with CK, FUNGuild predicted that OMM significantly increased the two trophic modes and 19 functional groups, and both saprotrophs and symbiotrophs significantly increased in the trophic modes (*p* < 0.05). Fungal parasite, wood saprotroph, undefined saprotroph, nematophagous, plant saprotroph, endophyte, litter saprotroph, soil saprotroph, and epiphyte were significantly up-regulated in the functional groups (*p* < 0.05) (Supplementary Table S14).

### 3.5 Mechanism underlying organic mulching materials for enhanced faba bean productivity

**Figure 6:**
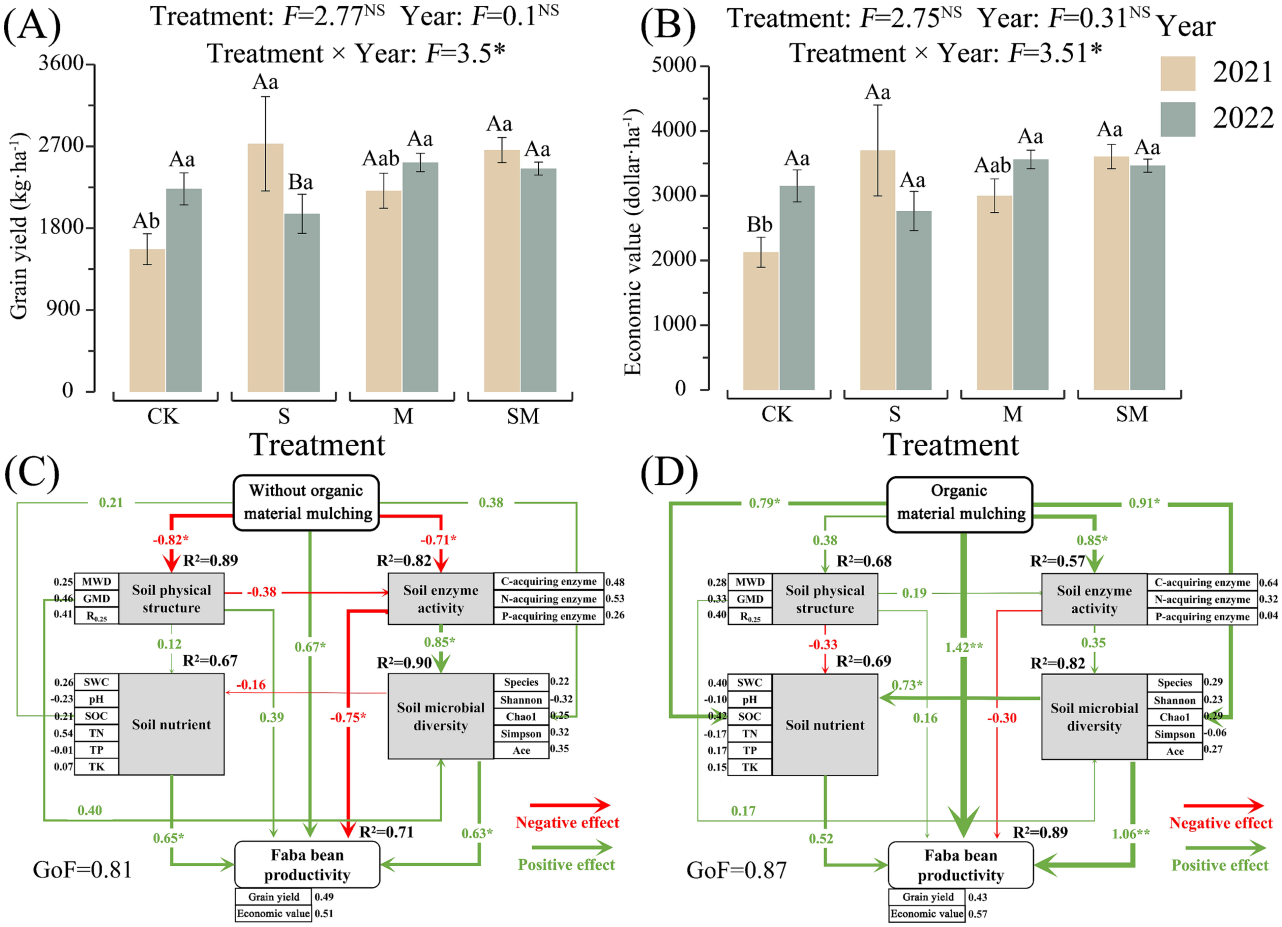
Faba bean productivity under CK and organic materials mulching treatments **(A,B)** and partial least squares path model (PLS-PM) illustrating the direct effects of **(C)** CK on the soil quality and grain yield of faba bean and **(D)** OMM (S, M, and SM) on the soil quality and grain yield of faba bean. Numbers on the arrowed lines and the thickness of the arrows indicate normalized path coefficients. Different lowercase letters indicate significant differences between different treatments of the same year. Different capital letters indicate significant differences between different years of the same treatment. CK, without organic material mulching; S, straw mulching; M, milk vetch mulching; SM, straw and milk vetch mulching.

In 2021, the grain yield and economic value of the faba bean in the OMM treatment field increased by 40.94–73.87% when compared with CK field and the increments observed in the case of S and SM treatment fields were highly significant (*p* < 0.05). In 2022, except for the S treatment field, the grain yield and economic value of the faba bean in the OMM treatment field increased by 9.95–12.99% when compared with CK field. The grain yield and economic value in M treatment fields increased with the increase in mulching time (Figures 6A,B).

The RFM analysis was performed using 17 abiotic and microbial variables (Supplementary Table S16). The results showed that no index had a significant effect on the grain yield of faba bean under the CK system. However, the changes in GMD, SOC, and Shannon index induced by OMM treatments had a significant positive effect on the grain yield of the faba bean (*p* < 0.05).

The goodness-of-fit (GOF) of the partial least squares path model (PLS-PM) for soil variables (abiotic and microbial) and faba bean grain yield was higher than 0.8 in CK as well as OMM treatments (Figures 6C,D), which confirms the reliability of the model. The PLS-PM indicated that predictor variables accounted for 68–89%, 67–69%, 57–82%, 82–90%, and 71–89% of the variations in the soil physical structure, soil nutrients, soil enzyme activity, soil microbial diversity, and faba bean productivity, respectively.

CK treatment significantly reduced the stability of soil physical structure (path coefficient = 0.89) and soil enzyme activity (path coefficient = 0.82), significantly increasing the grain yield of faba bean (path coefficient = 0.71) (*p* < 0.05). Further, soil enzyme activity significantly enhanced soil microbial diversity (path coefficient = 0.85), but significantly reduced the grain yield of faba bean (path coefficient = 0.75) (*p* < 0.05). The soil nutrients (path coefficient = 0.65) and soil microbial diversity (path coefficient = 0.63) had significant impacts on the productivity of the faba bean (*p* < 0.05) (Figure 6C).

OMM treatments significantly increased soil nutrients (path coefficient = 0.79), soil enzyme activity (path coefficient = 0.85), soil microbial diversity (path coefficient = 0.91), and faba bean grain yield (path coefficient = 1.42) (*p* < 0.05). Further, the increase in soil microbial diversity was associated with a significant increase in soil nutrients (path coefficient = 0.73) and faba bean productivity (path coefficient = 1.06) (*p* < 0.05).

## 4 Discussion

### 4.1 Mechanism underlying organic mulching materials for enhanced soil aggregates stability and carbon cycling efficiency of faba bean

The results of this study demonstrated that in 2021 and 2022, OMM treatments enhanced the SOC content and activities of the C acquisition enzyme when compared with CK. In 2022, OMM treatments significantly improved soil aggregate stability when compared with CK (*p* < 0.05). The stability of soil aggregates and SOC content increase with the increase in mulching time. These results indicated that the improvement in soil aggregates and C cycle by OMM treatments was a result of long-term accumulation. Returning organic materials to the field enhances soil aggregate stability and C cycling efficiency through interactions among aggregates, SOC content, C-acquisition enzymes, and microbial communities. Current research demonstrates that enhanced aggregate stability arises not only from organic-mineral coordination forming stable complexes, but also involves organic-induced adsorption of microbially-derived extracellular polymers (e.g., polysaccharides, proteins, humic substances), fungal hyphae, and colloidal materials, ultimately fostering microbial residue formation (Kan et al., 2022; Ai S. et al., 2023). Wang Y. et al. (2023) conducted a study for five consecutive years and reported that straw mulching can significantly improve the distribution and stability of soil aggregates. Kamran et al. (2021) performed long-term field experiments for 10 consecutive years and demonstrated that fresh milk vetch could significantly increase the distribution of large soil aggregates >0.25 mm.

The major reason for the improvement in soil aggregate stability through the return of organic materials to the field is the synergistic effect of abiotic and biotic activities in the soil, which is closely linked to the C cycle (Huang et al., 2017). In terms of abiotic components, SOC is a major contributor to several decomposition products that promote soil microbial activity, including carboxylic acids, alcohols, phenols, polysaccharides, and amines. These organic compounds bind with soil minerals and agglomerate to form microaggregates (Liu Y. et al., 2023). In this study, when compared with CK, OMM treatments effectively increased the SOC content (Supplementary Table S4). The RDA analysis demonstrated a positive correlation of OMM with SOC (Figures 5B,D). Numerous research studies have demonstrated that the return of straw and legume green fertilizer to the field has a significant influence on SOC and C mineralization (Hobbie and Hobbie, 2013; Dhaliwal et al., 2020). Soil biological activities manifest as variations in enzyme activity and microbial community structure, primarily derived from microorganisms, plants, animals, and organic residues (Sun et al., 2023). This study confirmed that the OMM treatments can promote the activity of key enzymes involved in the soil C cycle (Figure 5). Previous studies have demonstrated that the return of straw and legume green fertilizer to the field had a positive effect on enzyme activity associated with soil C cycle (Khan et al., 2020; Liu et al., 2022). Bowles et al. (2014) demonstrated that the application of legume green fertilizer can increase the activity of β-glucosidase and α-galactosidase, which are considered as key enzymes of the soil C cycle. Further, this study demonstrated the positive effect of OMM treatments on soil microbial diversity. Increased microbial diversity drives soil aggregation through mycelia production and polysaccharide secretion, enhancing aggregate formation and stabilization (Ren et al., 2018; Tsutsui et al., 2022). The microbial residues contain a large number of amino sugars, which are also important sources of SOC (Wang et al., 2021). In addition, the results of this study demonstrated a significant improvement in the relative abundance of *Bacteroidetes* and *Actinobacteria* by OMM treatments (Figure 3A). The metabolic energy of *Bacteroidetes* is derived from starch, cellulose, and other polysaccharide substances (Zheng et al., 2021). *Bacteroidetes*, as key decomposers mediating carbohydrate fermentation and nitrogenous organic compound utilization, drive straw decomposition processes (Tlaskal and Baldrian, 2021). *Actinobacteria* are capable of degrading cellulose and chitin, which are the major sources of soil nutrients (Ren et al., 2022). Further, this study revealed that the dominant bacterial groups in OMM treatment soil were *Alphaproteobacteria* and *Gammaproteobacteria* (Supplementary Figure S3; Supplementary Table S12). These two groups of bacteria can have a significant role in straw degradation and promote the accumulation of organic carbon (Ma et al., 2024). The RDA analysis confirmed a positive correlation of *Bacteroidetes*, *Acidobacteria*, *Ascomycota*, and *Basidiomycota* with C-acquiring enzyme activity and SOC (Figure 5).

### 4.2 Mechanism underlying organic mulching materials for enhanced soil nitrogen cycling efficiency of faba bean

OMM treatments elevated soil nitrogen content and N-acquisition enzyme activity relative to CK during 2021–2022, demonstrating enhanced coupling of soil carbon and nitrogen cycling. Straw serves as a key organic substrate providing C and energy for soil microorganisms, while enhancing the mineralizable organic N: available C ratio to stimulate soil N mineralization and nitrification (Chen et al., 2017). Several research studies have demonstrated that soil N mineralization and nitrification can increase with the increase in SOC content (Yu H. et al., 2022). This study revealed that the SOC, TN, and AN content in the S treatment field were higher than those in CK throughout the experiment in 2021 and 2022. The RDA confirmed a positive correlation between C and N contents and their enzyme activities, which revealed that SOC input could promote N cycling in dry farmlands to a certain extent (Supplementary Table S4; Figure 5). M treatment enhanced soil nitrogen cycling via milk vetch incorporation, a leguminous source supplying N–P–K-micronutrients and mediating symbiotic N fixation for nutrient enrichment (Ma et al., 2020). The results of this study demonstrated that S treatment could effectively improve soil N nutrient and N acquiring enzyme activity when compared with CK. In addition, RDA also confirmed a strong positive correlation of S with TN content and N-acquiring enzyme activity (Supplementary Table S4; Figure 5).

However, in this study, SM treatment outperformed other treatments in improving the efficiency of C and N cycling. Fresh straw decomposition depletes soil available nitrogen via microbial mineralization, impeding overall organic materials decomposition through induced N limitation (Hicks et al., 2021). Although the simple application of milk vetch is beneficial for N storage, it is not beneficial for the long-term enhancement of SOC (Zhong et al., 2021). Research studies have demonstrated that organic materials with a low C:N ratio are conducive to the decomposition of straw C by soil microorganisms. The decomposition products bind with soil minerals and contribute to the formation of aggregates (Zhang et al., 2017). SM treatment stimulated SOC accumulation through enhanced fresh straw decomposition and alleviated microbial mineral N competition via legume application (Xie et al., 2022a). These results provided evidence that SM treatment enhances SOC, TN, AN, and the activities of C and N acquiring enzymes in the soil (Supplementary Table S4; Figure 2). Straw-milk vetch co-incorporation stimulated microbial community diversification, yielding abundant polysaccharides that drive aggregate cementation through metabolic processes and enhance macroaggregate formation for C–N adsorption sites (Kamran et al., 2021). The findings of this study demonstrated that the major microbial class in the SM-enriched soil was *Anaerolineae* (Supplementary Table S6), a group of Gram-negative bacteria that can effectively degrade humus in the soil (Zhang et al., 2025). Research studies have demonstrated that *Anaerolineae* can reduce N fertilizer application under the influence of straw returning to the field, thus enriching the soil with this bacterial class and increasing the accumulation of nutrients (Zhang et al., 2019; Podosokorskaya et al., 2013).

### 4.3 Regulation of core microbial groups by organic mulching materials

The soil microbial community is the major component of the soil that mediates the cycling of important nutrients such as C, N, and P through anabolic and catabolic processes (Liu S. et al., 2023). The results of this study indicated that the OMM treatments can increase the structural and functional diversity of the soil microbial communities. The addition of organic materials can provide necessary nutrients and energy for microbial activities, and also increase the diversity of soil bacteria and fungi (Adetunji et al., 2020; Ren et al., 2021). Long-term straw return stimulates soil bacterial/fungal proliferation and microbial community richness, with microbial-derived amino sugars enhancing SOC accumulation and aggregate formation (Li, 2022), which is consistent with the results of this study. Straw with low density and light texture can increase the accumulated temperature and water retention capacity of the soil (Feng et al., 2021). Straw-milk vetch co-incorporation enhances macroaggregate formation and stability by supplying microbial membrane C reservoirs and metabolic energy, while fostering microbial colonization through habitat optimization and species richness elevation (Asghar and Kataoka, 2022; Yuan et al., 2023). These studies explain the enrichment of soil microorganisms through OMM treatments in this research. Kim et al. (2020) reported that OMM is a sustainable method to enhance agricultural production and has a positive impact on soil microbial metabolism. PICRUSt and FUNGuild analyses revealed OMM treatment possible upregulation of functional pathways (genetic information processing, metabolism, organismal systems) enhancing soil metabolic potential, while elevated proportion of saprotrophs and symbiotrophs may be drove soil C–N cycling efficiency through synergistic functional contributions (Supplementary Tables S13, S14). These findings are consistent with the previous studies demonstrating that organic materials return to the field can enhance the soil microbial community structure, promote co-trophic bacteria, and contribute to soil sustainability (Zhao et al., 2017; Price et al., 2021). In addition, this study demonstrated that the positive impact of SM treatment on soil microbial diversity was significantly higher than that of S and M treatments. This may be due to the higher P acquiring enzyme activity and soil P content in SM treatment, and the lower C:P acquiring enzyme activity ratio and N:P acquiring enzyme activity ratio. The greater soil P metabolic efficiency resulting from SM treatment may increase P availability for microbial energy production and enhance the microbial community structure (Lv et al., 2022).

Microbial MENs construction elucidates soil microbial community architecture and interaction dynamics through topological feature analysis (e.g., node/link influence), identifying keystone taxa driving community structure–function relationships (Ai Z. M. et al., 2023). This study demonstrated that OMM treatments enhanced the topological metrics of microbial MENs, including total nodes, connections, average degree, and modularity (Supplementary Tables S7, S8). These findings indicate an increase in the complexity and stability of microbial system architecture. Straw return to field enhanced soil co-occurrence network complexity in Xu et al. (2023), manifested through elevated connectivity, keystone taxa abundance, and topological metrics (average degree, clustering coefficient) reinforcing microbial network stability. In addition, the dominant species and microbial groups often play a vital role in maintaining the stability of microbial community structure and function (Marshall and Lynch, 2020). In this study, OMM treatments led to the establishment of a microbial community with *Actinobacteria*, *Bacteroidetes*, *Ascomycota*, and *Basidiomycota* as the core microbial phyla. These microorganisms play an active role in soil nutrient cycling and maintain the large energy consumption of the microbial community. It is worth mentioning that the dominant microbes of CK and OMM treatment soils belong to the phylum *Ascomycota*, which plays a vital role in the decomposition of soil organic materials. However, it requires a large amount of N as an energy source (Lopez-Mondejar et al., 2018). OMM can provide more N to soil compared with CK, which is one of the main reasons for the complexity of soil microbial MENs following the application of OMM.

### 4.4 Enhanced faba bean productivity by organic mulching materials through the promotion of soil microbial diversity and carbon cycling

The results of this study demonstrated that the OMM treatments could improve the yield and economic value of faba bean, which is consistent with the conclusion that straw mulching and purple milk vetch return to the field could improve crop yield (Wang M. et al., 2023). Since the variations of yield and economic value are the same, this study analyzed the effects of OMM on the faba bean yield alone when compared to CK using RFM analysis. The analysis results indicated that GMD, SOC, and Shannon index had significant effects on the faba bean grain yield under OMM treatments. There was a gradual decrease in the influence of SOC, C acquiring enzyme activity, N acquiring enzyme activity, and Shannon and Simpson indices on the grain yield of faba bean. These results indicated that SOC and microbial diversity were the major driving factors for the enhanced faba bean grain yield under the OMM treatments. Several studies have demonstrated that straw mulching enhanced soil active C pool and SOC content, thereby providing favorable conditions for growth and development of crops (Liu D. et al., 2023; Wang M. et al., 2023). Gai et al. (2019) reported that straw return to the field could improve crop yield by augmenting the direct nutrient supply for increasing soil fertility. Soil biodiversity elevation stimulates beneficial microorganism proliferation and interpopulation coordination, thereby improving soil quality and environmental stress resilience as demonstrated by Stefan et al. (2021). Soil microbial diversity stimulates crop yield through functional enhancement, with meta-analysis by Shu et al. (2022) demonstrating significant positive correlations among diversity, community structure, functionality, and productivity. In this study, PLS-PM demonstrated that OMM treatments significantly improved the soil nutrient content, soil microbial diversity, and faba bean productivity. OMM treatments enhanced soil nutrient availability and faba bean yield via soil microbial diversity-driven mechanisms, establishing microbial diversity as the primary driver under such amendments. The yield of S and SM treatments decreased during 2021–2022, with S exhibiting significant interannual variation contrasting SM stability. This divergence likely originated from S-mediated straw return without N supplementation, elevating soil organic carbon but inducing long-term microbial-crop N competition that reduced yields. Conversely, SM with N supplementation mitigated such competition. Strategic integration of optimized N inputs with organic-mineral fertilizers is recommended to maximize ecological benefits (Li et al., 2024; Yu et al., 2024).

Corn straw mulching revealed superior efficacy in enhancing soil quality and crop yield versus granulator-produced pellet incorporation, offering novel strategies for optimizing straw incorporation practices as demonstrated by Fan and Wu (2020). No-tillage combined with straw returning during conservation tillage plays an important role in promoting soil aggregate stability, soil C and N cycling, and microbial diversity (Guo et al., 2016). Further studies can be conducted to explore the ecological benefits of OMM treatments combined with no-tillage mode on farmland soils and crops. Future OMM treatment research should prioritize multi-omics integration for key C/N metabolic gene mapping and soil metabolome profiling, addressing current methodological gaps. These studies will be essential for a systematic understanding of the potential role of OMM in enhancing the ecological benefits of soil and crops in farmlands.

## 5 Conclusion

In this study, organic mulching materials (OMM) significantly enhanced soil macroaggregate stability and stimulated carbon (C) and nitrogen (N) accrual through elevated enzymatic activities over time. OMM treatment induced microbial community diversification with complex co-occurrence networks. The dominant phyla (Actinobacteria, Bacteroidetes, Ascomycota, Basidiomycota) exhibited strong positive correlations with C and N contents, as well as with nutrient-acquiring enzyme activities. The functional prediction analysis conducted using PICRUSt and FUNGuild suggests that the enhancement of soil metabolic capacity may be related to the processing, metabolism, and improvement of biological system pathways associated with microbial functional gene information. Furthermore, the increase in the proportion of saprotrophs and symbiotrophs may have improved the efficiency of soil C and N cycling. These microbial-driven mechanisms, particularly diversity indices followed by soil organic content (SOC) and C-acquiring enzymes activities, directly enhanced faba bean productivity and economic value. Our findings establish straw-milk vetch co-mulching as a sustainable strategy for augmenting agroecosystem services through simultaneous soil health improvement and yield optimization.

## Data Availability

The datasets presented in this study can be found in online repositories. The names of the repository/repositories and accession number(s) can be found in the article/Supplementary material.
