# Supplementary material for: Organic material mulching regulated core microbial groups to promote soil carbon and nitrogen cycling and improve faba bean productivity under a triple-cropping system in purple soil hilly region of southwest China
Source: Front Microbiol. 2025 Jul 16;16:1602633. doi: 10.3389/fmicb.2025.1602633 (PMC12307408; doi:10.3389/fmicb.2025.1602633)
Supplement: Supplementary file 1 [file Data_Sheet_1.docx]

Organic material mulching regulated core microbial groups to promote soil carbon and nitrogen cycling and improve faba bean productivity under a triple-cropping system in purple soil hilly region of southwest China

Ke Ren^1^, Wenfeng Song^1^, Zehui Wei^2^, Lixia Song^1^, Ming Liu^1^, Yuling Zhou^1^, Yuzhuo Zhen^1^, Xinyao Wu^1^, Kaiyuan Gu^1^, Khanom Simarani^2^* and Longchang Wang^1^*

^a^ College of Agronomy and Biotechnology, Southwest University / Engineering Research Center of South Upland Agriculture, Ministry of Education, 400715 Chongqing, China.

^b^ Division of Microbiology, Institute of Biological Sciences, Faculty of Science, Universiti Malaya, 56100 Kuala Lumpur, Malaysia.

*** Corresponding authors.** E-mail addresses: wanglc2003@163.com (L. C. Wang)**;** E-mail addresses: [hanom_ss@um.edu.my](mailto:hanom_ss@um.edu.my) (K. Simarani)

**Type of article:** Original research article

**Number of words:** 8464 words in the main text Number of figures and tables: 0 table and 6 figures **Supporting information:** 16 tables and 8 figures

**Supplementary methods**

**2.2.4 Soil DNA extraction and PCR amplification**

OTU Production: Sequences analysis were performed by Uparse software (Uparse v7.0.1001，<http://drive5.com/uparse/>) (Edgar, 2013). Sequences with ≥97% similarity were assigned to the same OTUs. Representative sequence for each OTU was screened for further annotation.

Species annotation: For each representative sequence, the Silva Database (<http://www.arb-silva.de/>) (Quast et al., 2012) was used based on Mothur algorithm to annotate taxonomic information.

Phylogenetic relationship Construction: In order to study phylogenetic relationship of different OTUs, and the difference of the dominant species in different samples (groups), multiple sequence alignment were conducted using the MUSCLE software (Version 3.8.31，http://www.drive5.com/muscle/)(Edgar, 2004).

Data Normalization: OTUs abundance information were normalized using a standard of sequence number corresponding to the sample with the least sequences. Subsequent analysis of alpha diversity and beta diversity were all performed basing on this output normalized data.

**2.7 Supplementary information on statistical analysis**

Genera with average relative abundances >0.1% were selected (Mao et al., 2022). By using the molecular ecological network analyses pipeline (MENAP) (http://ieg4.rccc.ou.edu/MENA) of the random matrix theory (RMT) constructs the co-occurrence network of soil microbial communities under CK and organic mulch materials treatments (Deng et al., 2012). At the same time, build 100 randomly rewire the network connections and calculate the network properties. Combined with the modularity threshold >0.4, it is used to judge whether the network meets the characteristics of "small world", "scale-free" and "modularity", which provides reliability support for subsequent network interaction analysis(Luo et al., 2014). The OTUs are used as the centered log-ratio transform (CLR) and similarity matrix-spearman's rho to construct the network structure. Select cutoff=0.9 to analyze network global properties, individual nodes' centrality, module separation and modularity calculation, poisson distribution < 0.05 by chi-square test is reserved. Obtain total nodes, total links, r square of power-law, average degree (avgK), average path distance (GD), average clustering coefficient (avgCC), node degree, modularity and other network parameters (Tolosana-Delgado, 2008). Network topology is characterized by intra-module connectivity (*Zi*) and inter-module connectivity (*Pi*) (Guimera and Amaral, 2005), which is mainly used to screen key species in the network. *Zi* measures the degree of "good connection" between node *i* and other nodes in the same module. The higher the value, the greater the role it plays in the module. *Pi* measures the connectivity between node *i* and other modules. A higher value indicates a closer relationship between node *i* and other modules. According to Zhou et al, they are divided into peripheral nodes (Peripherals, *Zi* < 2.5, *Pi*≤0.62), module hubs (Module hubs, *Zi*≥2.5, *Pi*≤0.62), network hubs (*Zi*≥2.5, *Pi* > 0.62), connectors (*Zi* < 2.5, *Pi* > 0.62) (Zhou et al., 2011). The key species in the co-occurrence network can be screened by connectors, module hubs and network hubs combined with node. stress and node. degree (Ju and Zhang, 2015; Wu et al., 2021).

In this study, Gephi 0.9.7 was used for modular analysis and visualization of the network based on the algorithm proposed. Colors of nodes signify different modules. Size of nodes signify different degrees(Huang et al., 2019).

By using the ranking analysis of canonical correspondence analysis (CCA) and redundancy analysis (RDA) functions in the vegan package, the r^2^ and P values of the effects of each environmental factor on species distribution can be calculated by the envfit function, and then the environmental factors with significant effects can be screened out. The bioenv function in the package can be used to screen out the environmental factors or combinations with the largest spearman correlation with species matrix, and then the selected environmental factors can be analyzed by CCA or RDA. Variance inflation (VIF) uses the vif. cca function in the package to screen the environmental factors with redundant constraints, and gradually eliminate the factors with VIF > 20 until all factors are less than 20. Then, non-redundant environmental factors are used for CCA and RDA analysis. Variance partial analysis (VPA) belongs to the partial method. RDA (*X, Y, Z*) in the vegan package is used to analyze the effects of main environmental factors (*Y*) and co-environmental factors (*Z*) on species distribution (*X*). It can quantify the amount of explanation for species distribution by certain environmental factors. The linear discriminant analysis effect size (LEfSe) was performed to identify the differentially abundant species of the specific bacterial communities.

To identify the major factors influencing grain yield of faba bean, we used random forest model (RFM) analysis(Breiman, 2001), wherein the relative importance of no organic mulch materials and organic mulch materials, Soil physical structure (MWD, GMD, R_0.25_) ,Soil nutrient (SWC, pH, SOC, TN, TP, TK), Soil enzyme activity (C acquiring enzyme, N acquiring enzyme, P acquiring enzyme), Soil microbial diversity (observed species, Shannon, chao1, Simpson, ace) were ranked. RFM analysis was done by “randomForest” package (Liaw and Wiener, 2002) and “rfPermute” package(Archer, 2020) in the R statistical computing environment. Additionally, we assessed the significance of the models cross validated R^2^ with 500 permutations of response variables using A3 package(Fortmann-Roe, 2015) in R 4.1.2.

Partial least squares path model (PLS-PM) was performed using the 'plspm' R package to further infer potential direct and indirect effects of CK and organic mulch materials treatments on soil environment and faba bean productivity.

Soil physical structure, soil nutrient, soil enzyme activity, soil microbial diversity and faba bean productivity are latent variables. Soil physical structure is indicated by MWD, GMD, and R_0.25_. Soil nutrient is indicated by SWC, pH, SOC, TN, TP, TK, and CEC. Soil enzyme activity is indicated by C acquiring enzyme, N acquiring enzyme, and P acquiring enzyme. Soil microbial diversity is indicated by Species, Shannon, Chao1, Simpson, and Ace. Faba bean productivity is indicated by grain yield and Output value. The goodness of fit (GoF) index is a pseudo-Goodness of fit measure that accounts for the model quality at both the measurement and the structural models. GoF can be used a global criterion that helps us to evaluate the performance of the model in both the inner and the outer models. Basically, GoF assess the overall prediction performance of the model. Acceptable “good” values within the PLS-PM community are GoF >0.7 (Sanchez, 2013). Loadings are calculated as correlations between a latent variable and its indicators Loadings greater than 0.7 are acceptable. They represent the amount of variability explained by a latent variable. A loading greater than 0.7 means that more than 0.7^2^ ≈ 50% of the variability in an indicator is captured by its latent construct.

**Supplementary Tables and Figures**

# Table captions:

**TABLE S1** Experimental treatment of faba bean-corn-sweet potato intercropping model in the multi-cropping system.

**TABLE S2** Nutrient content of different crops straw and milk vetch.

**TABLE S3** Distribution of soil mechanically stable aggregates under CK and organic mulch materials treatments.

**TABLE S4** Soil nutrient content under CK and organic mulch materials treatments.

**TABLE S5** The ɑ-diversity indices of soil bacterial communities under CK and organic mulch materials treatments.

**TABLE S6** The ɑ-diversity indices of soil fungal communities under CK and organic mulch materials treatments.

**TABLE S7** Characteristic parameters of the molecular ecological network of bacterial communities under CK and organic mulch materials treatments.

**TABLE S8** Characteristic parameters of the molecular ecological network of fungal communities under CK and organic mulch materials treatments.

**TABLE S9** Linear discriminant analysis effect size (LEfSe)Linear discriminant analysis (LDA) discriminant result chart of LDA score (log10) distribution.

**TABLE S10** Topological roles of individual module nodes and keystone species in the bacterial microbial network.

**TABLE S11** Topological roles of individual module nodes and keystone species in the fungal microbial network.

**TABLE S12** Screening the key species of molecular ecological network based on the topology role of individual module node.

**TABLE S13** The KEGG biological metabolic pathway of soil bacteria in CK and organic mulch materials treatments.

**TABLE S14** The functional and guild of soil fungi in CK and organic mulch materials treatments.

**TABLE S15** Key parameters of redundancy analysis (RDA) between soil microbial community structure and soil environmental factors.

**TABLE S16** The main predictors of the effect of CK and organic mulch materials system on the grain yield of faba bean. The table shows the random forest mean predictor importance (the percentage of increase in the mean variance error [MSE]) of abiotic and microbial variables on grain yield of faba bean for CK and organic mulch materials system.

# Figure Captions:

**FIGURE S1** Effects of CK and organic mulch materials treatments on soil aggregate stability indices.

**FIGURE S2** Heat map of clustering distribution of soil bacteria (A) and fungi (B) at the phylum and class level.

**FIGURE S3** Topological roles of individual module nodes and key species of soil bacteria and fungi in molecular ecological networks.

## Tables

**TABLE S1** Experimental treatment of "faba bean/corn/sweet potato" planting mode of triple-cropping system.

| Code | Treatment | Agronomic operation |
| --- | --- | --- |
| CK | Control check (without organic mulching materials) | No organic mulch materials treatment |
| S | Straw mulching | After harvest of each crop, the straw is cut into about 15cm in length and evenly covered on the soil surface. The straw cover amount of faba bean and corn is 7250 kg·ha, and the straw cover amount of sweet potato is 4500 kg·ha. |
| M | Milk vetch mulching | In the growth period of faba bean, the milk vetch is covered, and the milk vetch is harvested in the full flowering period and uniformly covered on the soil surface. |
| SM | Straw and milk vetch mulching | The straw mulching operation is the same code S, and the milk vetch mulching operation is the same code M. |

**TABLE S2** Nutrient content of different crops straw and milk vetch.

| Organic materials | Organic matter（g·kg^-1^） | Total nitrogen（g·kg^-1^） | Total phosphorus（g·kg^-1^） | Total potassium（g·kg^-1^） | Ammonium nitrogen（mg·kg^-1^） | Nitrate nitrogen（mg·kg^-1^） | Available phosphorus（mg·kg^-1^） | Available potassium（mg·kg^-1^） |
| --- | --- | --- | --- | --- | --- | --- | --- | --- |
| Corn straw | 783.06 | 7.94 | 0.97 | 16.63 | 445.26 | 2678.69 | 376.72 | 16.62 |
| Sweet potato straw | 690.5 | 13.01 | 3.12 | 22.54 | 195.41 | 1784.36 | 413.97 | 21.43 |
| Faba bean straw | 746.8 | 11.67 | 0.89 | 4.12 | 103.45 | 2686.18 | 803.17 | 3.48 |
| milk vetch | 754.61 | 25.58 | 3.3 | 25.65 | 191.5 | 3575.86 | 728.68 | 24.67 |

**TABLE S3** Distribution of soil mechanically stable aggregates under CK and organic mulch materials treatments.

| Year | Treatment | *R*_0.25_ (%) | ＞2000μm (%) | 250μm-2000μm (%) | 106μm-250μm (%) | ＜106μm (%) |
| --- | --- | --- | --- | --- | --- | --- |
| 2021 | CK | 82.64±0.85Aa | 46.79±0.92Aa | 35.85±1.77Aa | 9.57±0.26Aa | 7.79±0.59Aa |
|  | S | 83.41±2.21Aa | 48.43±1.8Aa | 34.99±0.84Aa | 9.91±1.13Aa | 6.67±1.11Aa |
|  | M | 82.11±0.29Ba | 44.76±2.31Ba | 37.35±2.4Aa | 9.53±0.41Aa | 8.36±0.13Aa |
|  | SM | 82.56±1.3Ba | 49.7±4.55Aa | 32.86±3.27Aa | 9.09±0.66Aa | 8.35±0.66Aa |
| 2022 | CK | 80.77±0.87Ab | 46.62±1.03Ab | 34.15±0.68Aab | 9.74±0.44Aa | 9.49±0.49Aa |
|  | S | 86.52±2.46Aa | 48.16±5.22Ab | 38.36±2.82Aa | 6.83±1.08Bb | 6.65±1.38Ab |
|  | M | 89.04±1.79Aa | 57.83±5.47Aa | 31.21±3.68Ab | 5.43±0.83Bb | 5.53±0.96Bb |
|  | SM | 88.14±1.55Aa | 58.76±4.14Aa | 29.38±2.6Ab | 5.8±0.78Bb | 6.06±0.79Ab |
| Factor (Df) | | *F* (*P*) | | | | |
| Year (1) | | 9.52(**) | 4.46(*) | 1.29(NS) | 22.91(**) | 2.06(NS) |
| Treatment (3) | | 2.66(NS) | 1.68(NS) | 1.77(NS) | 3.71(*) | 2.17(NS) |
| Year × Treatment (3) | | 3.03(NS) | 1.71(NS) | 1.32(NS) | 3.06(*) | 3.07(NS) |

Values are means ± SE (n = 3). Different lowercase letters indicate significant differences between different treatments of the same year. Different capital letters indicate significant differences between different years of the same treatment. “*” Indicates significantly different at 0.05 probability level. “**” Indicates significantly different at 0.01 probability level, and ns indicates no significant correlation at 0.05 probability level. The same below.

**TABLE S4** Soil nutrient content under CK and organic mulch materials treatments.

| Year | Treatment | SWC (%) | pH | SOC (g·kg^-1^) | TN (g·kg^-1^) | TP (g·kg^-1^) | TK (g·kg^-1^) | AN (mg·kg^-1^) | AP (mg·kg^-1^) | AK (mg·kg^-1^) |
| --- | --- | --- | --- | --- | --- | --- | --- | --- | --- | --- |
| 2021 | CK | 11.82±1.56Ba | 6.45±0.1Aa | 12.65±1.03Aa | 1.17±0.07Ac | 0.82±0.02Aa | 20.59±0.21Ba | 95.3±5.06Ab | 65.01±10.22Aa | 201.27±1.59Ab |
|  | S | 11.13±2.35Ba | 6.07±0.12Ac | 13.19±0.39Aa | 1.42±0.03Aab | 0.81±0.02Aa | 20.72±0.18Ba | 109.42±6.49Aab | 81.22±5.99Aa | 223.4±2.97Aab |
|  | M | 13.54±3Ba | 6.2±0.14Abc | 13.64±0.32Aa | 1.52±0.06Aa | 0.87±0.03Aa | 20.77±0.08Ba | 124.21±10.91Aa | 97.06±12.19Aa | 230.7±8.38Aa |
|  | SM | 14.93±1.28Ba | 6.25±0.24Aab | 14.12±0.57Ba | 1.3±0.07Abc | 0.84±0.06Aa | 20.53±0.03Ba | 110±22.39Aab | 94.33±36.24Aa | 218.17±16.79Aab |
| 2022 | CK | 21.11±1.01Aa | 6.06±0.04Ba | 12.68±1.04Ab | 1.23±0.03Aa | 0.92±0Aa | 21.97±0.07Ab | 115.16±12.47Ab | 55.78±7.47Aa | 198.87±14.73Ab |
|  | S | 24.17±0.57Aa | 6.28±0.02Aa | 14.74±0.83Aa | 1.25±0.09Ba | 0.89±0.04Aa | 22.31±0.07Aab | 125.12±4.92Aa | 58.7±0.52Aa | 211.2±0.97Aab |
|  | M | 22.88±1.14Aa | 5.98±0.07Aa | 14.11±0.46Aa | 1.34±0.02Ba | 0.9±0.03Aa | 22.67±0.14Aa | 127.61±4.32Aa | 56.89±6.2Aa | 206.73±10.38Aab |
|  | SM | 22.14±0.17Aa | 6.11±0.08Aa | 16.03±0.3Aa | 1.35±0.05Aa | 0.9±0.06Aa | 22.29±0.22Ab | 137.86±6.71Aa | 62.91±13.49Aa | 227.03±10.84Aa |
| Factor (Df) | | *F* (*P*) | | | | | | | | |
| Year (1) | | 70.98(**) | 2.56(NS) | 4.27(NS) | 2.15(NS) | 5.61(*) | 277.46(**) | 4.79(*) | 5.69(*) | 1.09(NS) |
| Treatment (3) | | 0.62(NS) | 0.63(NS) | 4.19(*) | 5.21(*) | 0.26(NS) | 3.39(*) | 1.50(NS) | 0.59(NS) | 1.98(NS) |
| Year × Treatment (3) | | 1.11(NS) | 2.23(NS) | 0.85(NS) | 2.54(NS) | 0.27(NS) | 1.27(NS) | 0.45(NS) | 0.37(NS) | 0.97(NS) |

**TABLE S5** The ɑ-diversity indices of soil bacterial communities under CK and organic mulch materials treatments.

| Year | Treatment | Observed species | Shannon | Chao1 | Simpson | Ace |
| --- | --- | --- | --- | --- | --- | --- |
| 2021 | CK | 1730.33±15.93Ab | 9.32±0.05Ab | 1919.93±26.02Aab | 0.996±0Ab | 1895.22±13.67Aab |
|  | S | 1606.33±76.78Bb | 9.18±0.08Ab | 1775.61±92.31Bb | 0.996±0Ab | 1759.25±83.55Bb |
|  | M | 1691±9.29Bb | 9.23±0.02Ab | 1902.78±17.07Bab | 0.996±0Ab | 1871.7±13.96Bab |
|  | SM | 1897.67±4.18Aa | 9.74±0.06Aa | 1998.34±7.67Aa | 0.997±0Aa | 2003.45±6.84Aa |
| 2022 | CK | 1675±27.84Ac | 9.15±0.04Ac | 1886.88±18.82Ab | 0.996±0Aa | 1871.44±20.45Ab |
|  | S | 1804±10.07Ab | 9.71±0.04Ab | 1921.88±21.61Ab | 0.997±0Aa | 1904.42±18.85Ab |
|  | M | 1962.67±30.3Aa | 9.87±0.04Aa | 2082.06±31.56Aa | 0.998±0Aa | 2069.61±29.69Aa |
|  | SM | 1990.67±1.76Aa | 9.93±0.01Aa | 2091.79±4.42Aa | 0.998±0Aa | 2078.59±6.39Aa |
| Factor (Df) | | *F* (*P*) | | | | |
| Year (1) | | 31.93(**) | 75.2(**) | 13.09(**) | 21.46(**) | 17.09(**) |
| Treatment (3) | | 26.41(**) | 52.15(**) | 10.88(**) | 12.55(**) | 15.1(**) |
| Year × Treatment (3) | | 9.99(**) | 28.07(**) | 3.06(NS) | 7.44(**) | 4.04(*) |

**TABLE S6** The ɑ-diversity indices of soil fungal communities under CK and organic mulch materials treatments.

| Year | Treatment | Observed species | Shannon | Chao1 | Simpson | Ace |
| --- | --- | --- | --- | --- | --- | --- |
| 2021 | CK | 702.33±39.41Aa | 3.7±0.35Aa | 830.03±32.93Aa | 0.1±0.04Aa | 846.81±33.06Aa |
|  | S | 733.67±29.73Aa | 4.12±0.29Aa | 848.13±36.61Aa | 0.06±0.03Aa | 858.55±34.36Aa |
|  | M | 683.67±40.07Aa | 3.88±0.33Aa | 793.75±29.86Aa | 0.08±0.04Aa | 805.04±33.39Aa |
|  | SM | 723.67±54.03Aa | 3.67±0.51Aa | 854.39±50.4Aa | 0.12±0.07Aa | 866.15±47.03Aa |
| 2022 | CK | 712±42.88Aa | 4.56±0.16Aa | 822.82±56.9Aa | 0.03±0.01Aa | 798.62±35.3Aa |
|  | S | 805.33±29.24Aa | 4.19±0.33Aa | 914.37±25.6Aa | 0.07±0.04Aa | 916.12±24.49Aa |
|  | M | 786.33±31.29Aa | 4.08±0.2Aa | 903.38±38.76Aa | 0.06±0.02Aa | 916.42±42.95Aa |
|  | SM | 717.33±35.22Aa | 3.93±0.23Aa | 826.41±17.78Aa | 0.09±0.03Aa | 800.52±41.38Aa |
| Factor (Df) | | *F* (*P*) | | | | |
| Year (1) | | 2.66(NS) | 2.37(NS) | 1.71(NS) | 0.78(NS) | 0.28(NS) |
| Treatment (3) | | 0.97(NS) | 0.52(NS) | 0.73(NS) | 0.4(NS) | 1.23(NS) |
| Year × Treatment (3) | | 0.89(NS) | 0.59(NS) | 1.44(NS) | 0.36(NS) | 2.62(NS) |

**TABLE S7** Characteristic parameters of the molecular ecological network of bacterial communities under CK and organic mulch materials treatments.

| Year | Network indexes | CK | S | M | SM |
| --- | --- | --- | --- | --- | --- |
| 2021 | Total nodes | 214 | 205 | 206 | 220 |
|  | Total links | 1715 | 2096 | 1626 | 1822 |
|  | Average degree (avgK) | 16.03 | 20.45 | 15.79 | 16.56 |
|  | Average clustering coefficient (avgCC) | 0.69 | 0.68 | 0.7 | 0.69 |
|  | Average path distance (GD) | 6.85 | 6.67 | 6.91 | 6.9 |
|  | Harmonic geodesic distance (HD) | 4.05 | 3.5 | 4.02 | 4.04 |
|  | Centralization of degree (CD) | 0.05 | 0.1 | 0.04 | 0.04 |
|  | Density (D) | 0.08 | 0.1 | 0.08 | 0.08 |
|  | Module | 4 | 10 | 8 | 9 |
|  | Modularity | 0.64 | 0.61 | 0.73 | 0.73 |
|  | Positive correlation (%) | 53.24 | 51.53 | 50.55 | 50.99 |
|  | Negative correlation (%) | 46.76 | 48.47 | 49.45 | 49.01 |
| 2022 | Total nodes | 204 | 241 | 239 | 221 |
|  | Total links | 1638 | 2217 | 2502 | 1845 |
|  | Average degree (avgK) | 16.06 | 18.4 | 20.94 | 16.7 |
|  | Average clustering coefficient (avgCC) | 0.69 | 0.69 | 0.7 | 0.69 |
|  | Average path distance (GD) | 6.85 | 6.8 | 6.61 | 6.87 |
|  | Harmonic geodesic distance (HD) | 3.96 | 4.01 | 3.73 | 4.02 |
|  | Centralization of degree (CD) | 0.04 | 0.05 | 0.08 | 0.04 |
|  | Density (D) | 0.08 | 0.08 | 0.09 | 0.08 |
|  | Module | 3 | 6 | 9 | 8 |
|  | Modularity | 0.62 | 0.75 | 0.65 | 0.71 |
|  | Positive correlation (%) | 51.51 | 52.14 | 50.04 | 50.03 |
|  | Negative correlation (%) | 48.49 | 47.86 | 49.96 | 49.97 |

**TABLE S8** Characteristic parameters of the molecular ecological network of fungal communities under CK and organic mulch materials treatments.

| Year | Network indexes | CK | S | M | SM |
| --- | --- | --- | --- | --- | --- |
| 2021 | Total nodes | 90 | 117 | 95 | 92 |
|  | Total links | 314 | 571 | 504 | 494 |
|  | Average degree (avgK) | 6.98 | 9.76 | 10.61 | 10.74 |
|  | Average clustering coefficient (avgCC) | 0.67 | 0.71 | 0.68 | 0.62 |
|  | Average path distance (GD) | 9.62 | 5.43 | 4.02 | 2.96 |
|  | Harmonic geodesic distance (HD) | 4.5 | 3.47 | 2.87 | 2.42 |
|  | Centralization of degree (CD) | 0.06 | 0.09 | 0.07 | 0.14 |
|  | Density (D) | 0.08 | 0.08 | 0.11 | 0.12 |
|  | Module | 4 | 11 | 6 | 6 |
|  | Modularity | 0.67 | 0.7 | 0.68 | 0.63 |
|  | Positive correlation (%) | 57.01 | 52.89 | 51.79 | 51.01 |
|  | Negative correlation (%) | 42.99 | 47.11 | 48.21 | 48.99 |
| 2022 | Total nodes | 100 | 104 | 104 | 115 |
|  | Total links | 415 | 693 | 552 | 817 |
|  | Average degree (avgK) | 8.3 | 13.33 | 10.62 | 14.21 |
|  | Average clustering coefficient (avgCC) | 0.7 | 0.65 | 0.68 | 0.69 |
|  | Average path distance (GD) | 3.34 | 4.09 | 5.13 | 3.34 |
|  | Harmonic geodesic distance (HD) | 2.76 | 2.76 | 3.28 | 2.58 |
|  | Centralization of degree (CD) | 0.18 | 0.1 | 0.04 | 0.1 |
|  | Density (D) | 0.08 | 0.13 | 0.1 | 0.13 |
|  | Module | 7 | 9 | 6 | 7 |
|  | Modularity | 0.72 | 0.57 | 0.7 | 0.65 |
|  | Positive correlation (%) | 54.94 | 52.24 | 52.54 | 51.77 |
|  | Negative correlation (%) | 45.06 | 47.76 | 47.46 | 48.23 |

**TABLE S9** Linear discriminant analysis effect size (LEfSe) Linear discriminant analysis (LDA) discriminant result chart of LDA score (log10) distribution.

*<See Appendix B. Supplementary table.xlsx>*

**TABLE S10** Topological roles of individual module nodes and keystone species in the bacterial microbial network.

*<See Appendix B. Supplementary table.xlsx>*

**TABLE S11** Topological roles of individual module nodes and keystone species in the fungal microbial network.

*<See Appendix B. Supplementary table.xlsx>*

**TABLE S12** Screening the key species of molecular ecological network based on the topology role of individual module node.

*<See Appendix B. Supplementary table.xlsx>*

**TABLE S13** The KEGG biological metabolic pathway of soil bacteria in CK and organic mulch materials treatments.

*<See Appendix B. Supplementary table.xlsx>*

**TABLE S14** The functional and guild of soil fungi in CK and organic mulch materials treatments.

*<See Appendix B. Supplementary table.xlsx>*

**TABLE S15** Key parameters of redundancy analysis (RDA) between soil microbial community structure and soil environmental factors.

*<See Appendix B. Supplementary table.xlsx>*

**TABLE S16** The main predictors of the effect of CK and organic mulch materials system on the grain yield of faba bean. The figure shows the random forest mean predictor importance (the percentage of increase in the mean variance error [MSE]) of abiotic and microbial variables on grain yield of faba bean for CK and organic mulch materials system.

*<See Appendix B. Supplementary table.xlsx>*

## Figure legends

**
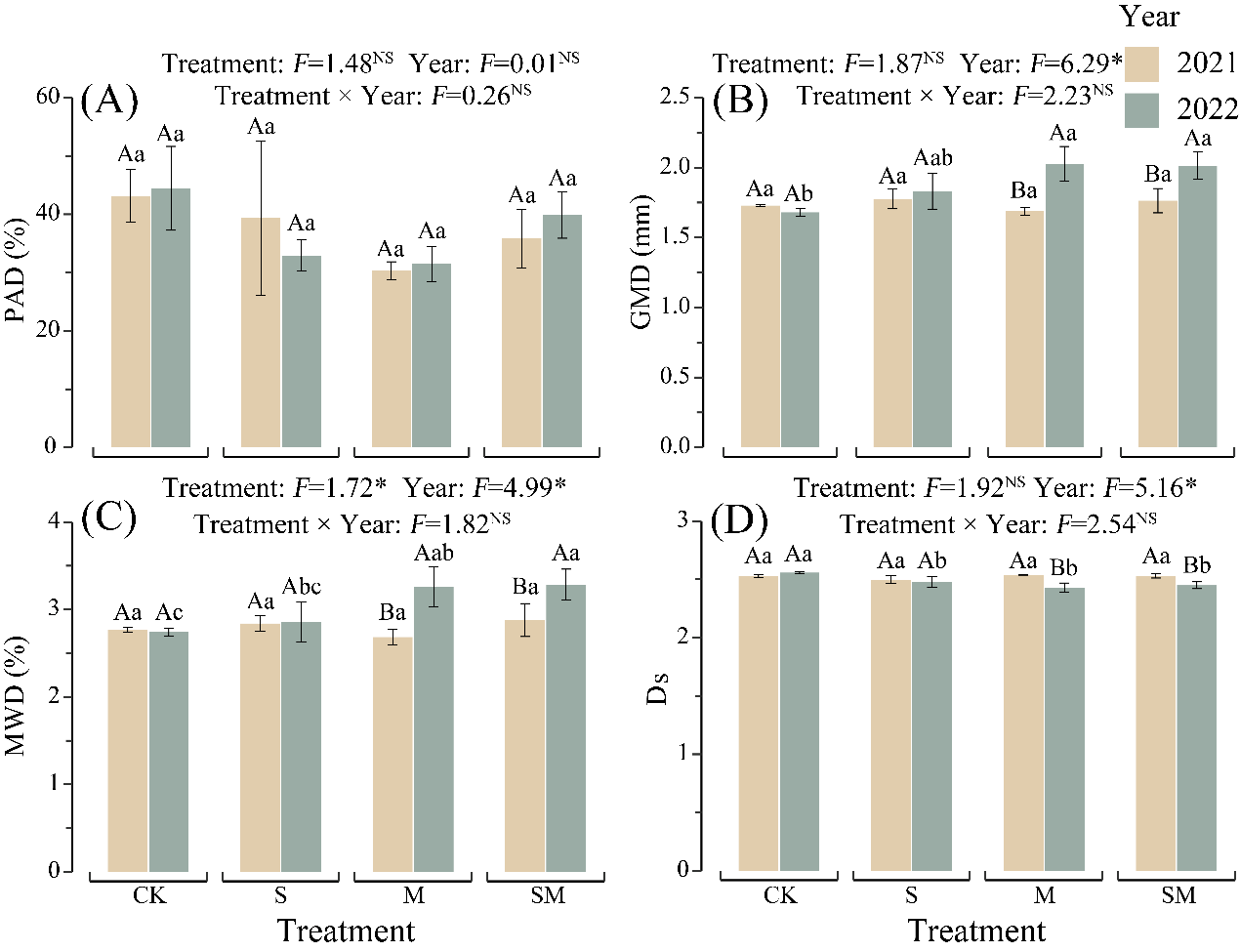
**

**FIGURE S1** Effects of CK and organic mulch materials treatments on soil aggregate stability indices.

(A) Percentage of aggregate destruction (PAD). (B) Geometric mean diameter (GMD). (C) Mean weight diameter (MWD). (D) Fractal dimensions (Ds). Values are mean ± standard error (SE) (n =3). Different lowercase letters indicate significant differences between different treatments of the same year. Different capital letters indicate significant differences between different years of the same treatment. CK, without organic material mulching; S, straw mulching; M, milk vetch mulching; SM, straw and milk vetch mulching.


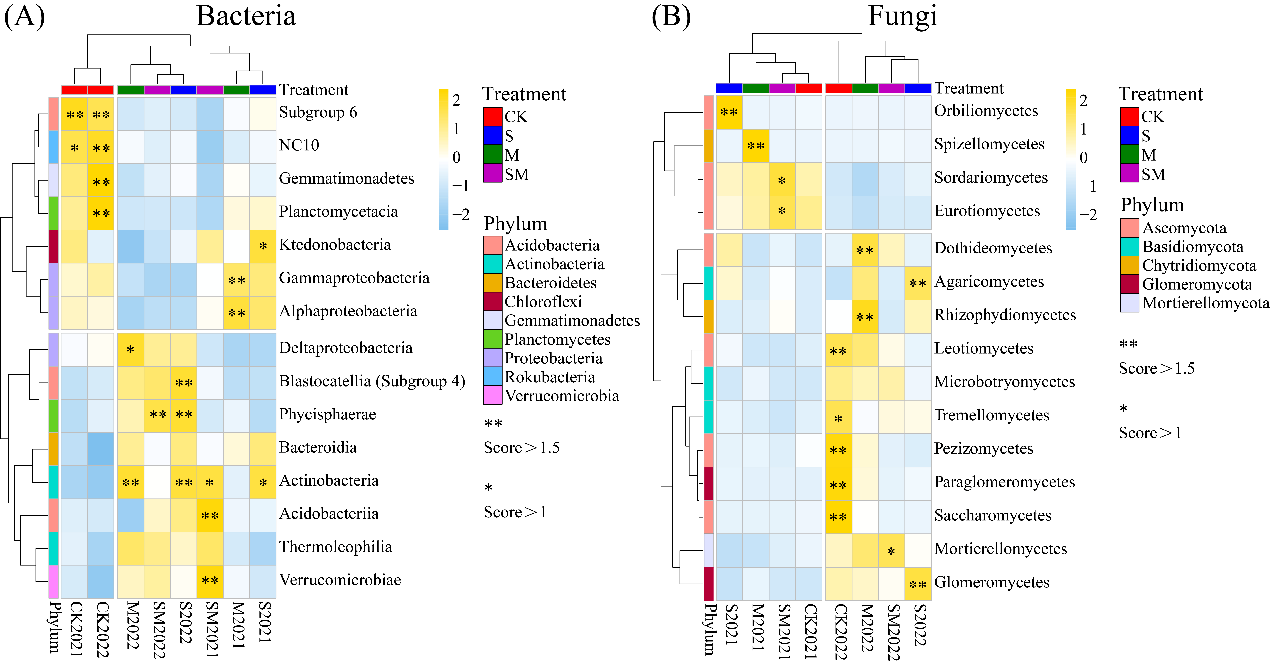


**FIGURE S2** Heat map of clustering distribution of soil bacteria (A) and fungi (B) at the phylum and class level. CK, without organic material mulching; S, straw mulching; M, milk vetch mulching; SM, straw and milk vetch mulching.

**
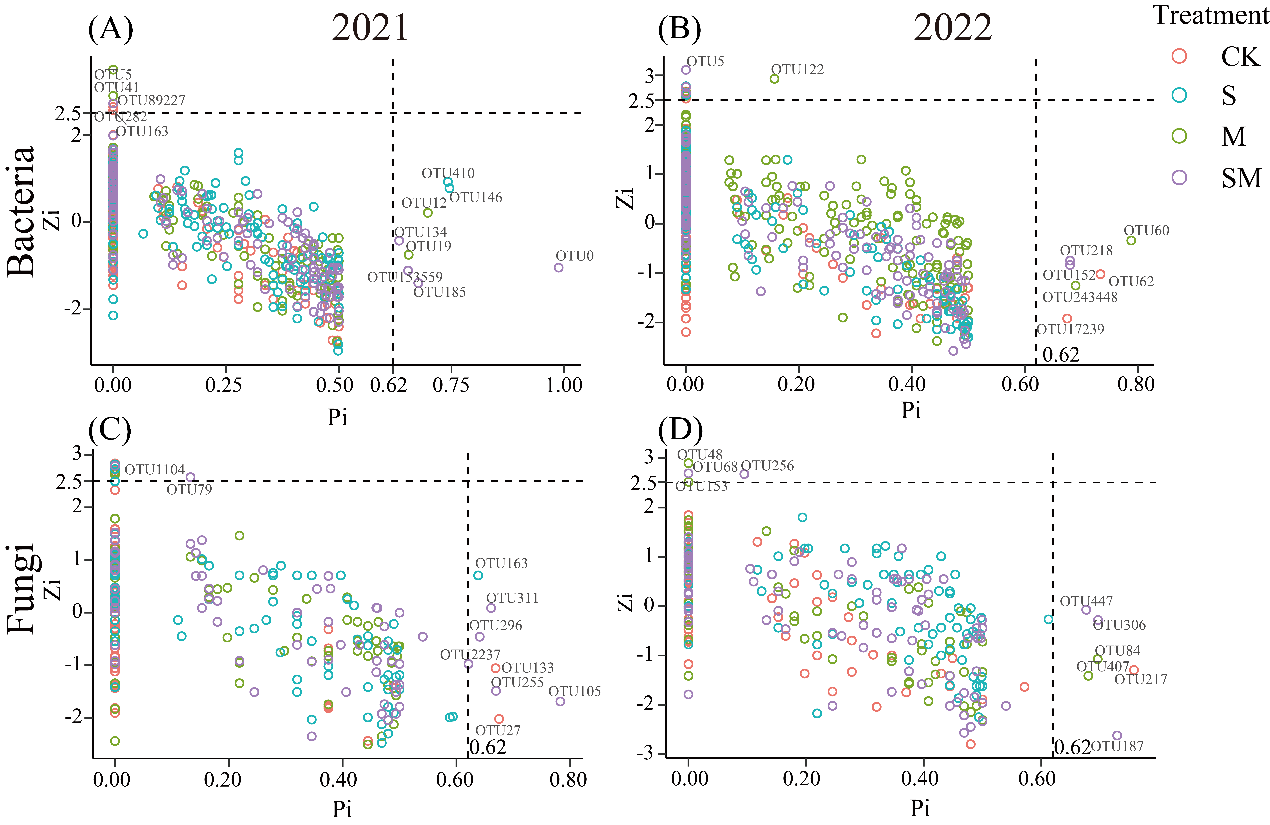
**

**FIGURE S3** Topological roles of individual module nodes and key species of soil bacteria and fungi in molecular ecological networks. (A, B) Key species of bacterial biomarkers in soil samples obtained from 2021 and 2022 fields, respectively. (C, D) Key species of fungal biomarkers in soil samples obtained from 2021 and 2022 fields, respectively. CK, without organic material mulching; S, straw mulching; M, milk vetch mulching; SM, straw and milk vetch mulching.

**References**

Archer, E. (2020). Rfpermute: Estimate permutation p-values for ran dom forest important metrics. R package version 2.2. https://CRAN.R-project.org/package=rfPermute.

Breiman, L. (2001). Random forests. *Machine Learning* 45(1)**,** 5-32. doi: 10.1023/A:1010933404324.

Deng, Y., Jiang, Y.-H., Yang, Y., He, Z., Luo, F., and Zhou, J. (2012). Molecular ecological network analyses. *BMC Bioinf.* 13(1)**,** 113. doi: 10.1186/1471-2105-13-113.

Edgar, R. C. (2004). MUSCLE: Multiple sequence alignment with high accuracy and high throughput. *Nucleic Acids Res.* 32(5)**,** 1792-1797. doi: 10.1093/nar/gkh340.

Edgar, R. C. (2013). UPARSE: Highly accurate OTU sequences from microbial amplicon reads. *Nat. Methods* 10(10)**,** 996-998. doi: 10.1038/nmeth.2604.

Fortmann-Roe, S. (2015). Consistent and clear reporting of results from diverse modeling techniques: The a3 method. *J. Stat. Software* 66(7)**,** 1-23. doi: 10.18637/jss.v066.i07.

Guimera, R., and Amaral, L. A. N. (2005). Cartography of complex networks: Modules and universal roles. *J. Stat. Mech.: Theory Exp.* 2005(02)**,** P02001. doi: 10.1088/1742-5468/2005/02/P02001.

Huang, R., Zhang, Z., Xiao, X., Zhang, N., Wang, X., Yang, Z., et al. (2019). Structural changes of soil organic matter and the linkage to rhizosphere bacterial communities with biochar amendment in manure fertilized soils. *Sci. Total Environ.* 692**,** 333-343. doi: 10.1016/j.scitotenv.2019.07.262.

Ju, F., and Zhang, T. (2015). Bacterial assembly and temporal dynamics in activated sludge of a full-scale municipal wastewater treatment plant. *ISME J.* 9(3)**,** 683-695. doi: 10.1038/ismej.2014.162.

Leo Breiman, Adele Cutler, Andy Liaw, and Wiener, M. (2018). Breiman and cutler's random forests for classification and regression [Package ‘randomForest’ 4.6-14]. https://www.stat.berkeley.edu/~breiman/RandomForests/

Luo, C., Rodriguez-R, L. M., Johnston, E. R., Wu, L., Cheng, L., Xue, K., et al. (2014). Soil microbial community responses to a decade of warming as revealed by comparative metagenomics. *Appl. Environ. Microbiol.* 80(5)**,** 1777-1786. doi: 10.1128/AEM.03712-13.

Mao, X., Yang, Y., Guan, P., Geng, L., Ma, L., Di, H., et al. (2022). Remediation of organic amendments on soil salinization: Focusing on the relationship between soil salts and microbial communities. *Ecotoxicol. Environ. Saf.* 239**,** 113616. doi: 10.1016/j.ecoenv.2022.113616.

Quast, C., Pruesse, E., Yilmaz, P., Gerken, J., Schweer, T., Yarza, P., et al. (2012). The SILVA ribosomal RNA gene database project: Improved data processing and web-based tools. *Nucleic Acids Res.* 41(D1)**,** D590-D596. doi: 10.1093/nar/gks1219.

Tolosana-Delgado, R. (2008). Compositional data analysis in a nutshell. *University of Gottingen on-line reference*.

Wu, B., Wang, P., Devlin, A. T., Chen, L., Xia, Y., Zhang, H., et al. (2021). Spatial and temporal distribution of bacterioplankton molecular ecological networks in the yuan river under different human activity intensity. *Microorganisms* 9(7)**,** 1532. doi: 10.3390/microorganisms9071532

Zhou, J., Deng, Y., Luo, F., He, Z., and Yang, Y. (2011). Phylogenetic molecular ecological network of soil microbial communities in response to elevated CO_2_. *mBio* 2(4)**,** e00122-00111. doi: 10.1128/mbio.00122-11.
